# Crotonylome profiling identifies MLKL crotonylation in lupus nephritis associated with RAB1A–mTOR signalling and autophagy changes in tubular epithelial cells

**DOI:** 10.1136/lupus-2026-002116

**Published:** 2026-08-31

**Authors:** Rui-Ling Lu, Shu-Hui Meng, Quan-Zhou Peng, Yanran Chen, Xufa Yang, Hong yang Liu, Feng-Ping Zheng, Dongzhou Liu, Xiaoping Hong

**Affiliations:** 1 Department of Rheumatology and Immunology, Shenzhen People’s Hospital (The Second Clinical Medical College, Jinan University; The First Affiliated Hospital, Southern University of Science and Technology), Shenzhen, China; 2 Department of Biomedical Sciences, City University of Hong Kong, Hong Kong, Hong Kong; 3 Department of Pathology, Shenzhen People’s Hospital (The Second Clinical Medical College, Jinan University; The First Affiliated Hospital, Southern University of Science and Technology), Shenzhen, China

**Keywords:** Lupus Nephritis, Cytokines, Lupus Erythematosus, Systemic

## Abstract

**Objective:**

To investigate whether MLKL crotonylation is associated with tubular autophagy–lysosome pathway homeostasis in lupus nephritis (LN) and to explore its relationship with RAB1A–mechanistic target of rapamycin (mTOR) signalling.

**Methods:**

Crotonylome proteomics was performed in peripheral blood mononuclear cells from patients with LN, patients with systemic lupus erythematosus without nephritis and healthy controls. Renal biopsy tissues were evaluated for tubulointerstitial fibrosis and autophagy–lysosome pathway-related markers. Mechanistic studies were conducted in lipopolysaccharide-stimulated HK-2 cells. Autophagic flux was assessed using bafilomycin A1. The dependency of mTOR/autophagy changes on RAB1A was tested by siRNA-mediated knockdown.

**Results:**

MLKL was identified as a differentially crotonylated protein in LN, with increased crotonylation at K95 and K219. Kidney tissues from patients with LN showed increased fibronectin and collagen III deposition compared with controls, whereas no significant difference was observed between class IV and class V LN. LC3 signal did not differ significantly between groups, whereas LAMP1 expression and LC3–LAMP1 co-localisation were reduced in LN. In HK-2 cells, crotonylation-deficient MLKL mutants were associated with increased LC3-II and reduced p62, whereas K219Q showed the opposite pattern. Autophagic flux assays using bafilomycin A1 showed that K219R-expressing cells had higher LC3-II levels than WT cells both before and after lysosomal inhibition, with comparable BafA1-induced LC3-II accumulation, consistent with increased autophagosome formation rather than impaired lysosomal degradation. HDAC1 knockdown increased MLKL crotonylation and was accompanied by mTOR activation. MLKL crotonylation enhanced RAB1A guanriphosphat osphate (GTP) binding without altering total RAB1A abundance. RAB1A knockdown in MLKL WT-expressing cells attenuated mTOR phosphorylation and partly reversed the autophagy-suppressive marker profile. Sodium crotonate induced an autophagy-suppressive marker profile that was partly reversed by rapamycin.

**Conclusion:**

MLKL crotonylation is associated with activation of the RAB1A–mTOR axis and altered tubular autophagy–lysosome pathway homeostasis in LN. These findings suggest that tubular injury-related changes in LN may not be fully reflected by glomerulus-based classification alone.

**WHAT IS ALREADY KNOWN ON THIS TOPIC**

**WHAT THIS STUDY ADDS**

- MLKL was identified as a differentially crotonylated protein in LN, with increased crotonylation at K95 and K219.
- LN kidney tissues showed increased tubulointerstitial matrix remodelling and reduced LAMP1 expression and LC3–LAMP1 co-localisation.
- In tubular epithelial cells, MLKL crotonylation was associated with enhanced RAB1A GTP binding, mTOR activation and an autophagy-suppressive marker profile. These findings were further supported by autophagic flux assays and RAB1A knockdown experiments.

**HOW THIS STUDY MIGHT AFFECT RESEARCH, PRACTICE OR POLICY**

- These findings support a role for MLKL crotonylation in tubular injury-related pathways in LN.
- The findings suggest that tubular injury-related changes may not be fully captured by glomerular classification alone.
- This work provides a basis for future studies of crotonylation-related mechanisms and tubular-targeted therapeutic strategies in LN.

## Introduction

 Systemic lupus erythematosus (SLE) is a multisystem autoimmune disease characterised by dysregulated immune responses and heterogeneous clinical manifestations.^1^ Renal involvement develops in approximately 50% of patients with SLE,^2^ and lupus nephritis (LN) remains a major contributor to morbidity and mortality. Delayed diagnosis and treatment increase the risk of progression to end-stage kidney disease (ESKD).^2–4^ The pathogenesis of LN is complex and involves complement activation, leucocyte recruitment, innate immune activation and environmental triggers such as infection.^5 6^ Current treatment for LN still relies largely on immunosuppressive agents, including cyclophosphamide, azathioprine and mycophenolate mofetil. However, relapse remains common, underscoring the need for a better mechanistic understanding of LN and more targeted therapies.^7^

Post-translational modifications (PTMs) of lysine residues regulate protein function under both physiological and pathological conditions.^8^ Lysine crotonylation is a covalent PTM found on histone and non-histone proteins and can influence gene expression and intracellular signalling.^9^ Altered crotonylation has been reported in kidney injury and cystic kidney disease, suggesting disease-relevant roles in the kidney.^10 11^ However, its contribution to LN, particularly within the tubular compartment, remains unclear.

Autophagy is a conserved degradative pathway that maintains cellular homeostasis by recycling proteins and organelles.^12^ In the kidney, autophagy is critical for tubular cell function and adaptation to stress, and dysregulation of autophagy has been implicated in LN.^13^ The mechanistic target of rapamycin (mTOR) pathway is a key regulator of autophagy and inflammation.^14–16^ At the same time, accumulating evidence indicates that tubulointerstitial injury is a major determinant of renal outcome in LN, complementing glomerulus-centred classification systems.^17^ Nevertheless, the upstream mechanisms linking PTMs to mTOR-related pathways and tubular homeostatic processes in LN remain insufficiently characterised.

In this study, we focused on MLKL crotonylation and its potential role in LN. We found that MLKL crotonylation enhanced the GTP-binding activity of RAB1A, was associated with activation of mTOR signalling and was linked to suppression of autophagy-related readouts in renal tubular epithelial cells. These findings provide mechanistic insight into how MLKL crotonylation-mediated regulation of the RAB1A–mTOR axis may contribute to tubular homeostatic disturbance in LN.

## Methods

### Study design

This study integrated peripheral blood mononuclear cells (PBMC) crotonylome profiling, renal tissue assessment and mechanistic interrogation in tubular epithelial cells. Crotonylation profiling was performed in PBMCs from patients with LN, patients with SLE without nephritis and healthy controls, followed by validation of MLKL crotonylation and site mapping. Renal biopsies were evaluated for tubulointerstitial extracellular matrix remodelling and autophagy–lysosome pathway-related readouts. Mechanistic studies were then performed in lipopolysaccharide (LPS)-stimulated HK-2 cells using MLKL site-directed mutants, HDAC1 knockdown, protein–protein interaction analysis and pharmacological modulation of crotonylation and mTORC1.

### Participants and PBMC isolation

PBMCs were collected from patients with SLE and healthy volunteers after written informed consent. The study was approved by the Ethics Committee of Shenzhen People’s Hospital (LL-KY-2022476-02). SLE was classified according to the 2019 EULAR/ACR criteria. LN was defined as 24-hour urinary protein excretion of >0.5 g, with renal biopsy performed when clinically indicated. PBMCs were isolated from ethylenediaminetetraacetic acid (EDTA)-anticoagulated blood by Ficoll density-gradient centrifugation and stored at −80 °C until analysis.

### Renal tissue specimens and histology/immunostaining

Renal biopsy specimens were obtained from patients with LN. Control kidney tissues were collected from nephrectomy specimens by sampling renal cortex at least 5 cm away from the lesion. The PBMC and biopsy cohorts were partially overlapping but not identical. Clinical characteristics of the PBMC cohort are summarised in online supplemental table S2; those of the renal tissue cohort are summarised in online supplemental table S3. Tissues were fixed in formalin, embedded in paraffin and sectioned at 3–4 µm.

For immunohistochemistry (IHC), sections were deparaffinised, rehydrated and subjected to heat-induced antigen retrieval in citrate buffer (pH 6.0) or EDTA buffer (pH 9.0), according to the antibody datasheets. Endogenous peroxidase activity was quenched with 3% H₂O₂, and sections were blocked with 5% normal serum or 5% bovine serum albumin. Primary antibodies against fibronectin (FN) and collagen III (COL3) were incubated overnight at 4 °C, followed by HRP-conjugated secondary antibodies, 3,3'-diaminobenzidine (DAB) development and haematoxylin counterstaining.

For immunofluorescence (IF), sections underwent antigen retrieval and blocking as described above, followed by overnight incubation at 4 °C with primary antibodies against LC3 and LAMP1. Fluorophore-conjugated secondary antibodies were then applied, and nuclei were counterstained with 4′,6-diamidino-2-phenylindole (DAPI).

Tubulointerstitial regions of interest were defined morphologically, excluding glomeruli and large vessels. For each subject, at least three randomly selected non-overlapping fields were analysed, and the average value for each subject was used for statistical testing. IHC signals were quantified as positive area fraction (%). LC3 and LAMP1 signals, as well as LC3–LAMP1 co-localisation, were quantified after background subtraction and consistent thresholding. LC3–LAMP1 co-localisation was quantified using the Manders’ overlap coefficient. Image analysis was performed in a blinded manner.

### Protein extraction, digestion and crotonylated peptide enrichment

Samples were lysed in buffer containing 1% Triton X-100 and protease inhibitors, sonicated on ice and clarified by centrifugation at 12,000×g for 10 min at 4 °C. Protein concentration was measured by bicinchoninic acid (BCA) assay. Proteins were precipitated with trichloroacetic acid (final concentration 20%), washed with prechilled acetone and resuspended in 200 mM triethylammonium bicarbonate. Disulfide bonds were reduced with 5 mM dithiothreitol at 56 °C for 30 min and alkylated with 11 mM iodoacetamide at room temperature for 15 min in the dark. Trypsin digestion was performed overnight at an enzyme-to-protein ratio of 1:50 (w/w). Peptides were desalted using Strata X SPE columns.

For crotonylome enrichment, peptides were incubated overnight at 4 °C with anti-crotonyllysine antibody beads (PTM Bio; lot PTM503) in NETN buffer. This antibody has been validated and used in multiple published crotonylome studies. Beads were washed, and bound peptides were eluted with 0.1% trifluoroacetic acid, vacuum-dried and desalted with C18 ZipTips before liquid chromatography-tandem mass spectrometry (LC-MS/MS) analysis.

### LC–MS/MS acquisition and database search

LC-MS/MS analysis was performed by Jingjie PTM BioLab (Hangzhou, China). Peptides were analysed on a NanoElute UHPLC system coupled to a timsTOF Pro mass spectrometer operating in parallel accumulation serial fragmentation mode. Peptides were separated on a homemade reversed-phase analytical column (25 cm, 100 µm internal diameter) using a gradient of solvent A (0.1% formic acid, 2% acetonitrile) and solvent B (0.1% formic acid in acetonitrile) at a flow rate of 450 nL/min. The MS/MS scan range was 100–1700 m/z and dynamic exclusion was set to 24 s.

Raw data were processed with MaxQuant (V.1.6.15.0) against the Homo_sapiens_9606_SP_20230103.fasta database using a reverse decoy strategy. Trypsin/P was specified with up to four missed cleavages, a minimum peptide length of seven amino acids and a maximum of five modifications per peptide. Carbamidomethylation of cysteine was set as a fixed modification, whereas methionine oxidation, protein N-terminal acetylation and lysine crotonylation were set as variable modifications. Precursor and fragment mass tolerances were both set to 20 ppm. The false discovery rate was controlled at <1% at both the peptide and protein levels.

### Bioinformatics analysis

Protein domain annotation was performed using Pfam/PfamScan. Subcellular localisation was annotated using curated databases, including UniProt, and prediction tools when needed. Kyoto Encyclopedia of Genes and Genomes (KEGG) pathway enrichment analysis was performed using Fisher’s exact test with Benjamini–Hochberg correction, and adjusted p<0.05 was considered significant. Protein–protein interaction networks were constructed using the STRING database with a confidence threshold of 0.4.

### Cell culture, transfection and treatments

HK-2 cells were maintained in DMEM/F12 supplemented with 10% fetal bovine serum at 37 °C in 5% CO₂. Plasmids encoding MLKL wild-type (WT) and site-directed mutants (K95R, K219R, K95/219R, K95Q and K219Q) were transiently transfected using Lipofectamine 3000. LPS stimulation was performed at 5 µg/mL for 24 hours.^18^ To increase global crotonylation, cells were treated with sodium crotonate (NaCr, 8 mM) for 48 hours. To inhibit mTORC1, rapamycin was used at 18 ng/mL for 48 hours.^19 20^ siRNA targeting HDAC1 and RAB1A was synthesised commercially; sequences are listed in online supplemental table S4. For autophagic flux assessment, bafilomycin A1 (BafA1; 100 nM) was added during the final 4 hours of culture to block lysosomal degradation. Autophagic flux was estimated as ΔLC3-II=LC3-II(₊BafA1) − LC3-II(₋BafA1).^21^

### Immunoprecipitation/co-immunoprecipitation and immunoprecipitation–mass spectrometry (IP–MS)

Protein complexes were enriched using a Protein A/G magnetic bead kit. Cell lysates were incubated overnight at 4 °C with primary antibodies, including anti-crotonyllysine, anti-MLKL and anti-RAB1A, captured on beads, washed and eluted for immunoblotting. For IP-MS, immunoprecipitated complexes were reduced, alkylated, digested with trypsin at a 1:50 ratio for 16 hours, desalted and analysed on an Orbitrap Exploris 480. Data were processed using Proteome Discoverer 2.4.

### Western blotting and enzyme-linked immunosorbent assay (ELISA)

Cell lysates were prepared in RIPA buffer supplemented with trichostatin A (TSA) (3 µM), nicotinamide (NAM) (50 mM), protease inhibitors and phenylmethylsulfonyl fluoride (PMSF). Proteins were separated by sodium dodecyl sulfate‑polyacrylamide gel electrophoresis (SDS-PAGE), transferred to PVDF membranes and probed with the indicated antibodies (online supplemental table S5). Key immunoblot results were obtained from at least three independent biological replicates. Densitometric quantification was performed using ImageJ, with band intensities normalised to loading controls. ELISA kits were used to measure TNF-α, IL-6 and IL-1β in culture supernatants according to the manufacturers’ instructions.

### Cell viability and cell death assessment

Cell viability was measured by CCK-8 assay. HK-2 cells expressing MLKL WT or mutant constructs were seeded in 96-well plates, stimulated with LPS (5 µg/mL, 24 hours) and incubated with CCK-8 reagent for 2 hours. Absorbance was read at 450 nm. Cell death was assessed by Annexin V-APC/PI staining and flow cytometry. TNF-α stimulation was included to evaluate cell death under necroptosis-promoting conditions.

### RAB1A GTP-binding assay

GTP-agarose pull-down was used to assess the GTP-binding capacity of RAB1A.^22^ Cell lysates were incubated with GTP-agarose at 4 °C, washed and eluted for immunoblotting.

### Statistical analysis

**Figure 1:**
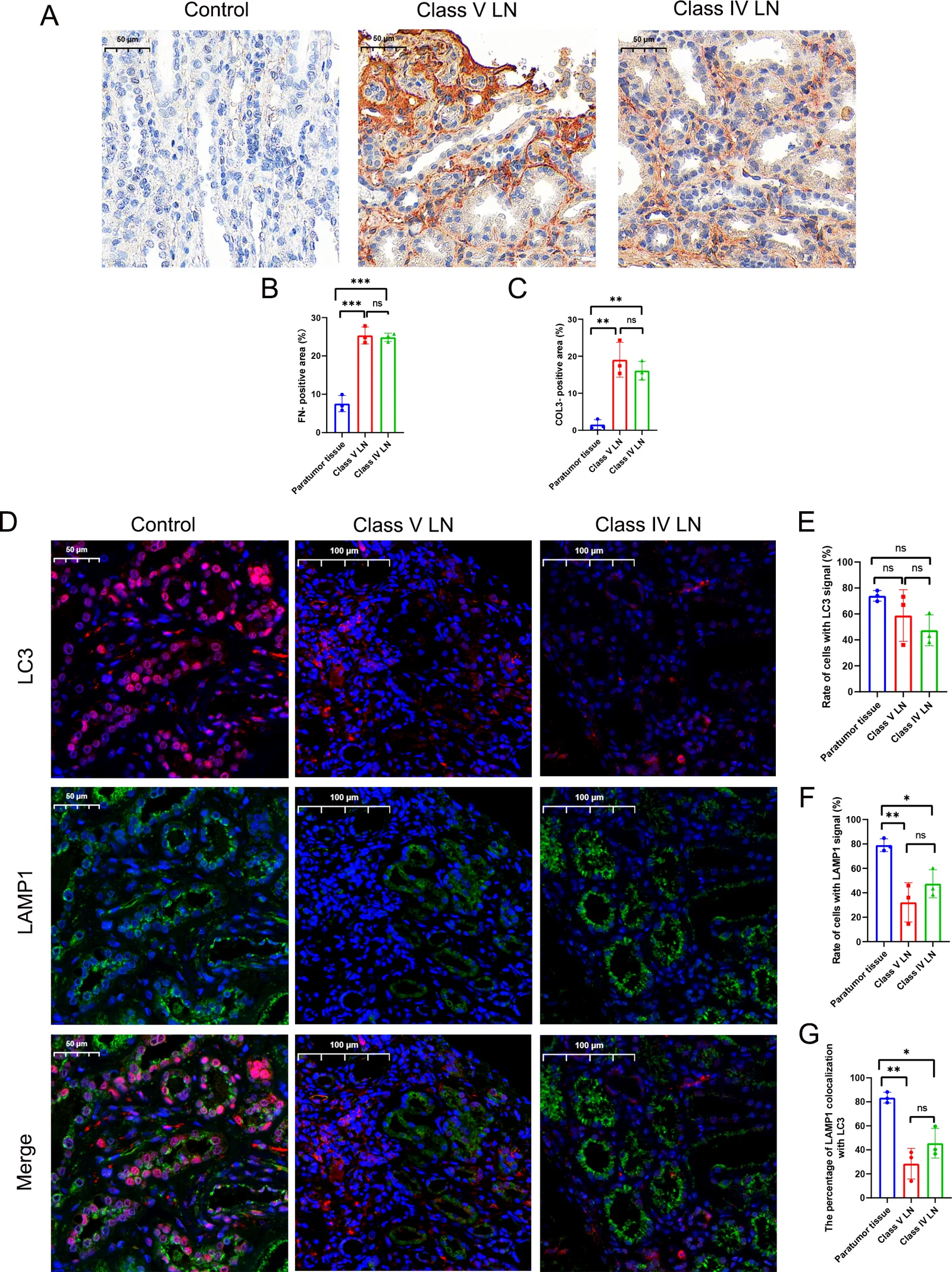
Renal biopsy assessment of tubulointerstitial fibrosis and autophagy–lysosome pathway markers in lupus nephritis. (**A**) Representative IHC images of FN and COL3 in control kidney tissues and LN renal biopsies. (**B**) Quantification of FN staining in control, class IV LN and class V LN groups. (**C**) Quantification of COL3 staining in control, class IV LN and class V LN groups. (**D**) Representative IF images of LC3 and LAMP1 in tubular regions. (**E**) Quantification of LC3 signal. (**F**) Quantification of LAMP1 signal. (**G**) Quantification of LC3–LAMP1 co-localisation. LC3 signal did not differ significantly among groups. LAMP1 expression and LC3–LAMP1 co-localisation were reduced in LN compared with controls, with no significant differences between class IV and class V LN. Scale bars, 50 µm. Data are presented as mean±SEM (n=3 per group). Statistical analysis was performed using one-way ANOVA with Tukey’s post hoc test. *p<0.05, **p<0.01. ANOVA, analysis of variance; COL3, collagen III; FN, fibronectin; IHC, immunohistochemistry; LN, lupus nephritis.

Statistical analyses were performed using GraphPad Prism V.8.0.2. Normality was assessed using the Shapiro–Wilk test. Comparisons between two groups were made using a two-sided unpaired t test or Mann–Whitney U test, as appropriate. Comparisons among multiple groups were performed using one-way analysis of variance (ANOVA) or the Kruskal–Wallis test, as appropriate. For figure 1, differences among control, class IV LN and class V LN were analysed using one-way ANOVA. A p value <0.05 was considered statistically significant. In autophagic flux experiments, ΔLC3-II values were compared between groups using an unpaired two-sided t test. For immunoblot densitometry, data from at least three independent experiments were analysed by one-way ANOVA with Tukey’s post hoc test or unpaired t test, as appropriate. Exact p values and the number of independent experiments are reported in the figure legends.

## Results

### PBMC crotonylome profiling identified widespread crotonylation changes in LN

**Figure 2:**
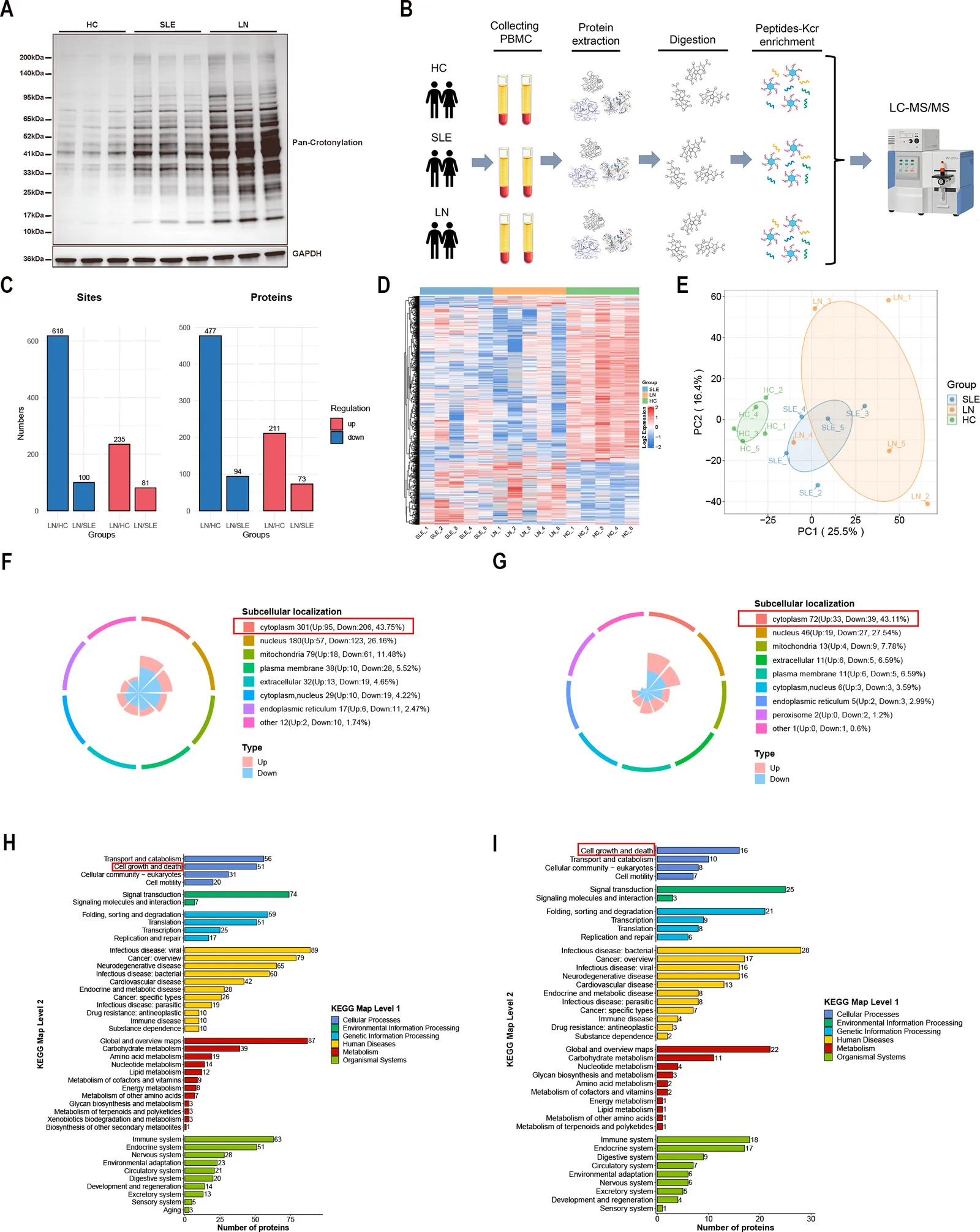
Identification and bioinformatics analysis of differentially crotonylated proteins and sites. (**A**) Western blot analysis of PBMCs from the three study groups using an anti-crotonyllysine antibody. (**B**) Schematic representation of crotonylome profiling in PBMCs using 4D label-free quantification. (**C**) Distribution of crotonylation sites across different abundance levels in the study groups. (**D**) Heatmap showing differences in crotonylated proteins between patient samples and healthy controls. (**E**) Principal component analysis showing separation among groups across datasets. (**F,G**) Subcellular distribution of crotonylated proteins in the LN group compared with the healthy control group and the SLE group. (**H,I**) KEGG functional classification of crotonylated proteins in the LN group compared with the healthy control group and the SLE group. HC, healthy control; LN, lupus nephritis; PBMC, peripheral blood mononuclear cell; SLE, systemic lupus erythematosus.

To define crotonylation changes associated with LN, PBMCs from patients with LN, patients with SLE without nephritis and healthy controls were analysed by pan-crotonyllysine immunoblotting and 4D label-free crotonylome proteomics (figure 2A,B). The identified peptides showed charge-state and length distributions consistent with efficient tryptic digestion and robust MS/MS performance (online supplemental figure S1A). Pearson correlation analysis showed high reproducibility across samples (online supplemental figure S1B). In total, 4455 proteins and 18 196 crotonylation sites were identified (online supplemental figure S1C).

Compared with healthy controls, LN samples showed 235 upregulated sites on 211 proteins and 618 downregulated sites on 477 proteins. Compared with SLE without nephritis, LN samples showed 81 upregulated sites on 73 proteins and 100 downregulated sites on 94 proteins (figure 2C–E). Differentially crotonylated proteins were enriched in the cytoplasm and in pathways related to cell growth and cell death (figure 2F–I).

### MLKL crotonylation was increased in PBMCs from patients with LN

**Figure 3:**
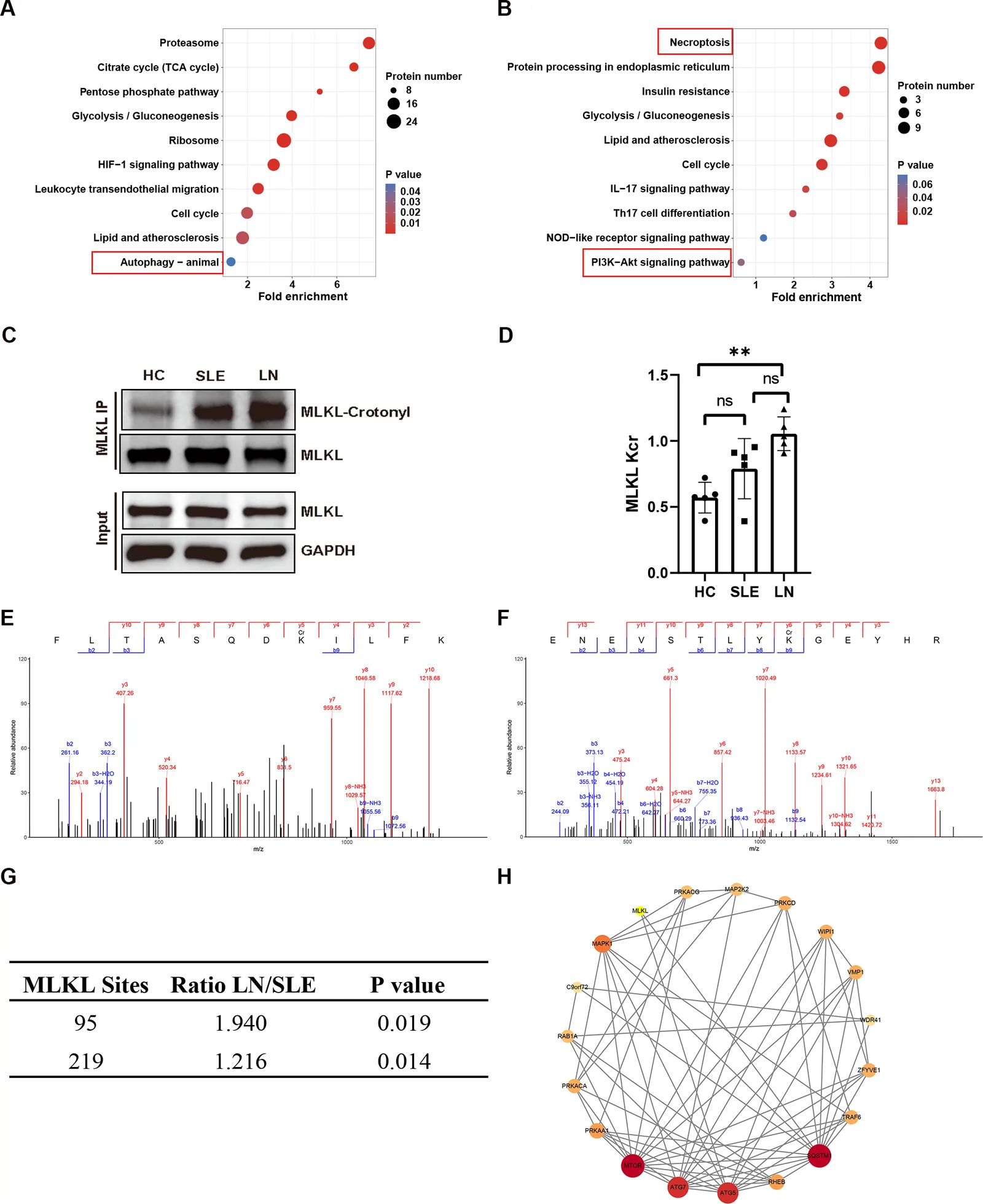
MLKL crotonylation was increased in lupus nephritis. (**A,B**) KEGG enrichment analysis of crotonylated proteins in the LN group compared with the healthy control group and the SLE group. (**C**) Quantification of MLKL crotonylation levels in PBMCs from the study groups. (**D**) Representative immunoprecipitation and immunoblotting results corresponding to (**C**). (**E,F**) Crotonylated MLKL at K95 and K219 identified in PBMCs from a patient with LN by mass spectrometry. (**G**) Relative abundance and p values for MLKL crotonylation at different sites between the LN and SLE groups. (**H**) Protein–protein interaction network involving MLKL and autophagy-associated proteins with altered crotonylation and/or protein expression. Data are presented as mean±SEM. *p<0.05, **p<0.01. LN, lupus nephritis; PBMC, peripheral blood mononuclear cell; SLE, systemic lupus erythematosus.

Pathway analysis highlighted autophagy-related pathways and showed stronger enrichment of the necroptosis pathway in LN than in SLE without nephritis (figure 3A,B), leading us to prioritise MLKL for further analysis. MLKL emerged as a necroptosis-related protein with increased crotonylation in LN. Immunoprecipitation of MLKL followed by pan-crotonyllysine immunoblotting confirmed higher MLKL crotonylation in LN PBMCs than in controls (figure 3C,D). LC–MS/MS site mapping identified lysine 95 (K95) and lysine 219 (K219) as the major crotonylation sites (figure 3E–G;online supplemental figure S2). A STRING-based interaction network of autophagy-associated proteins with altered crotonylation and/or expression placed MLKL in a connected node with multiple autophagy-related factors (figure 3H).

### Tubulointerstitial remodelling and altered autophagy–lysosome pathway markers were observed in LN kidneys

To assess renal relevance, we examined LN biopsy tissues and control kidney specimens. IHC showed increased FN and COL3 staining in the tubulointerstitial compartment of LN kidneys compared with controls (figure 1A–C), whereas no significant difference was detected between class IV and class V LN.

IF analysis showed that LC3 signal did not differ significantly between LN and control kidney tissues or between class IV and class V LN. By contrast, LAMP1 expression and LC3–LAMP1 co-localisation were reduced in tubular regions of LN kidneys compared with controls, with no significant difference between class IV and class V LN (figure 1D–G). These findings are consistent with altered autophagy–lysosome pathway homeostasis in the tubular compartment in LN.

### MLKL crotonylation status was associated with autophagy-related markers and cytokine production in tubular epithelial cells

**Figure 4:**
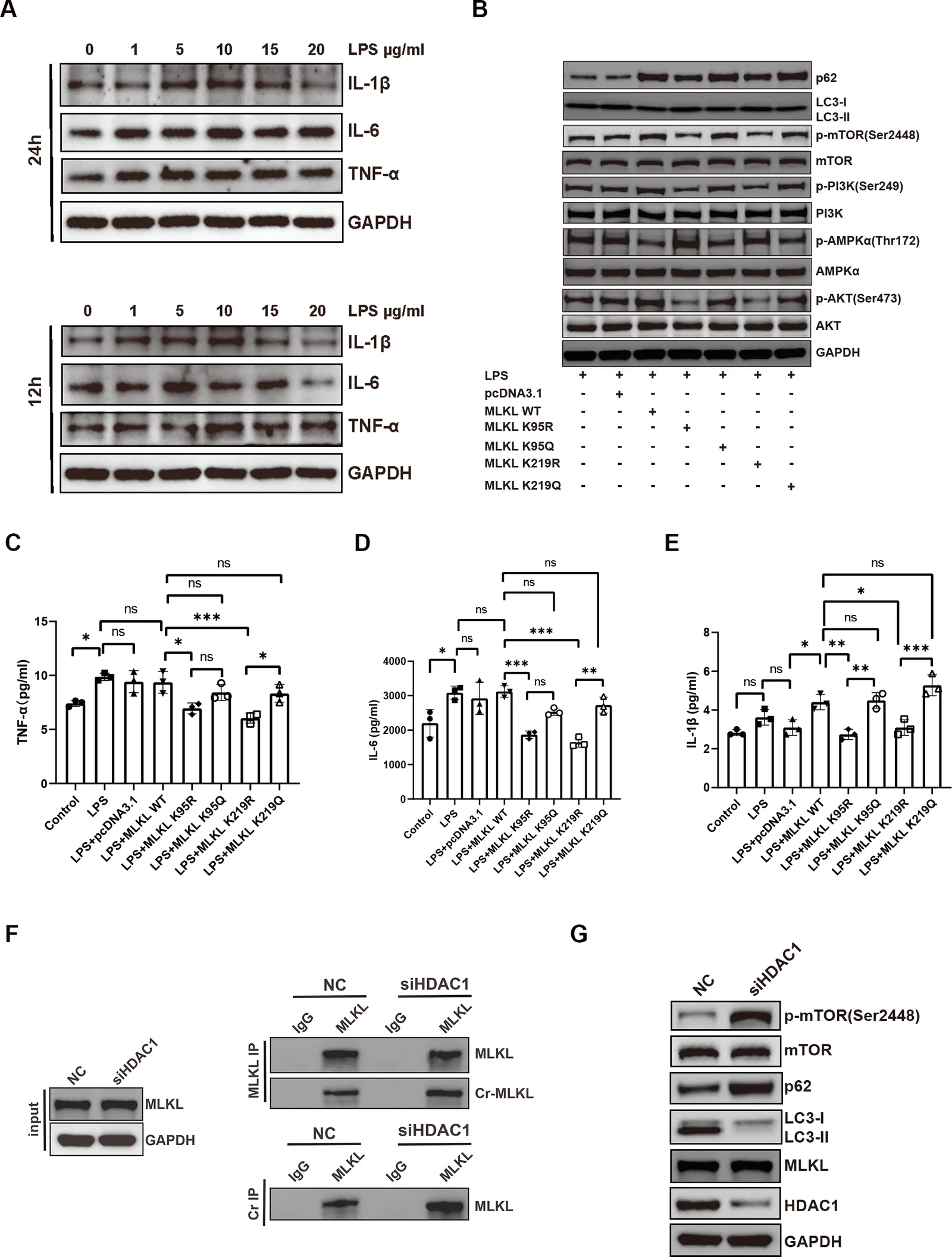
MLKL crotonylation status was associated with autophagy-related markers and inflammatory responses in HK-2 cells. (**A**) Western blot analysis of inflammatory markers in HK-2 cells stimulated with different concentrations of LPS for different durations. (**B**) Western blot analysis of autophagy-related proteins and signalling molecules in HK-2 cells expressing MLKL constructs with different crotonylation-related states. (C–E) ELISA measurement of TNF-α, IL-6 and IL-1β in HK-2 cells expressing MLKL constructs with different crotonylation-related states. (**F**) Immunoprecipitation and immunoblotting analysis of MLKL crotonylation in HK-2 cells transfected with siRNA-HDAC1 for 48 hours. (**G**) Western blot analysis of autophagy-related proteins in HK-2 cells transfected with siRNA-HDAC1 for 48 hours. One-way ANOVA was used for multiple-group comparisons. Data are mean±SEM from n=3 independent experiments. Unpaired t test. *p<0.05, **p<0.01, ***p<0.001. ANOVA, analysis of variance.

To model tubular innate immune activation, we used LPS-stimulated HK-2 cells. LPS induced a robust inflammatory response at 24 hours, and 5 µg/mL was used in subsequent experiments to limit potential cytotoxicity (figure 4A). To examine the contribution of specific crotonylation sites, we generated MLKL mutants with K-to-R substitutions (K95R, K219R and K95/219R) as crotonylation-deficient variants and K-to-Q substitutions as charge-neutralising putative acylation-related variants.

Under LPS stimulation, cells expressing crotonylation-deficient MLKL mutants (K95R, K219R) showed significantly reduced p62/SQSTM1 compared with MLKL WT. LC3-II was significantly increased in K219R-expressing cells compared with WT and showed a similar trend in K95R cells (figure 4B, online supplemental figure S3). The K95R mutant showed significantly reduced p-mTOR/mTOR and p-AKT/AKT ratios and increased p-AMPK/AMPK compared with WT. K219R showed a similar pattern, with significantly increased p-AMPK/AMPK and reduced p-AKT/AKT, while p-mTOR/mTOR showed a trend toward reduction (p=0.055). Changes in p-PI3K/PI3K did not reach statistical significance (figure 4B, online supplemental figure S3). The charge-neutralising K219Q variant did not differ significantly from WT in any of these readouts. ELISA showed that TNF-α, IL-6 and IL-1β levels were significantly lower in the K95R and K219R groups than in the WT group (figure 4C–E). Together, these data suggest that loss of MLKL crotonylation at K95 and K219 is associated with reduced p62, increased AMPK activation, attenuated AKT and mTOR signalling, and lower inflammatory cytokine production in LPS-stimulated tubular epithelial cells.

### Autophagic flux was enhanced in cells expressing crotonylation-deficient MLKL

Steady-state LC3-II and p62 levels alone cannot distinguish between enhanced autophagy and impaired lysosomal degradation.^21^ To address this, we performed autophagic flux assays using BafA1. In the absence of BafA1, K219R-expressing cells showed significantly higher LC3-II levels than WT cells (p=0.048). BafA1 treatment increased LC3-II in both groups, and K219R-expressing cells maintained higher LC3-II levels than WT cells after BafA1 treatment. The BafA1-induced ΔLC3-II was comparable between WT and K219R groups, indicating that the elevated LC3-II in K219R cells reflected increased autophagosome formation rather than impaired lysosomal degradation.^21^ Baseline p62 levels tended to be lower in K219R than in WT cells (p=0.052), and after BafA1 treatment, p62 levels did not differ significantly between the two groups, consistent with comparable lysosomal degradation capacity (online supplemental figure S4). The flux assay was not performed for K95R, which should be addressed in future work.

### HDAC1 knockdown increased MLKL crotonylation and was associated with mTOR activation

To examine enzymatic regulation of MLKL crotonylation, HK-2 cells were transfected with siRNA targeting HDAC1. MLKL immunoprecipitation followed by pan-crotonyllysine immunoblotting showed that MLKL crotonylation increased after HDAC1 knockdown (figure 4F). HDAC1 knockdown was also accompanied by increased p-mTOR and an autophagy-suppressive marker profile, characterised by increased p62/SQSTM1 and reduced LC3-II (figure 4G). These findings support a link between increased MLKL crotonylation, mTOR activation and suppression of autophagy-related markers. Because HDAC1 knockdown elevates global acylation levels across many substrates, these data show that HDAC1 regulates MLKL crotonylation, but the downstream mTOR and autophagy changes cannot be attributed to MLKL alone.

### MLKL crotonylation was associated with increased RAB1A activity

**Figure 5:**
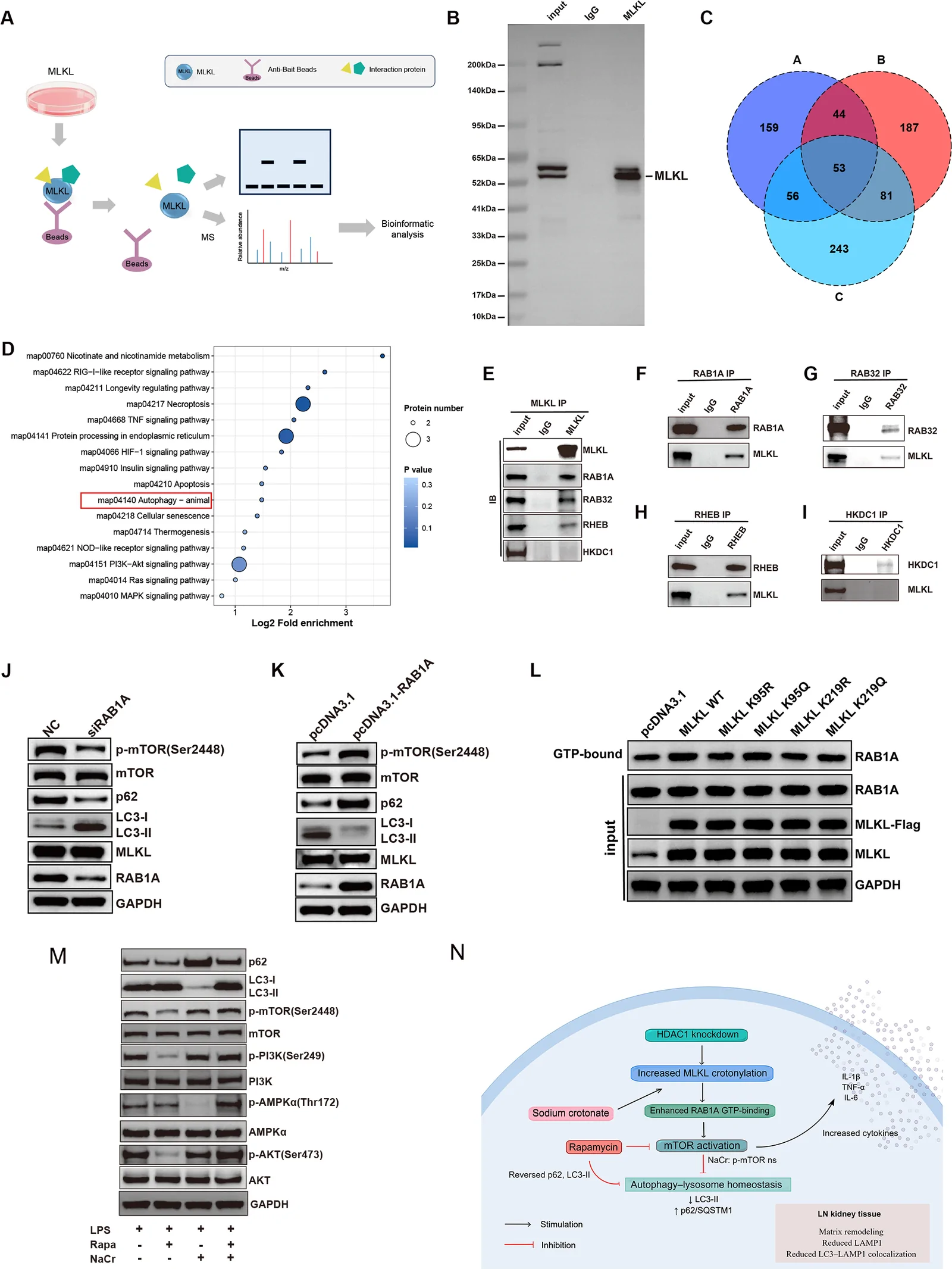
MLKL interacted with RAB1A and crotonylation-associated changes contributed to altered autophagy–lysosome homeostasis. (A–C) IP-MS was performed to identify the interacting partners of MLKL. (**D**) Kyoto Encyclopedia of Genes and Genomes enrichment analysis of proteins interacting with MLKL. (E–I) Co-IP assay showing the endogenous interaction between MLKL and RAB1A. (**J**) Western blot analysis of autophagy-associated proteins in HK-2 cells transfected with siRNA-RAB1A for 48 hours. (**K**) Western blot analysis of autophagy-associated proteins in HK-2 cells transfected with a RAB1A plasmid for 48 hours. (**L**) RAB1A GTP-binding assay in HK-2 cells expressing MLKL constructs under different Kcr-related conditions. (**M**) Western blot analysis of autophagy-associated proteins in HK-2 cells treated with 8 mM NaCr and/or 18 ng/mL rapamycin for 48 hours. (**N**) Proposed model summarising the findings of the present study. In tubular epithelial cells, HDAC1 knockdown was associated with increased MLKL crotonylation, enhanced RAB1A GTP binding and mTOR activation. These changes were accompanied by autophagy–lysosome alterations, including reduced LC3-II and increased p62/SQSTM1, as well as increased cytokine production. Sodium crotonate increased p62 and decreased LC3-II, although p-mTOR was not significantly changed (NaCr: p-mTOR ns, as indicated in the model). Rapamycin partly reversed the changes in p62 and LC3-II. In kidney tissues from patients with lupus nephritis, matrix remodelling, reduced LAMP1 expression and decreased LC3–LAMP1 co-localisation were observed, consistent with altered tubular autophagy–lysosome homeostasis. mTOR, mechanistic target of rapamycin.

To identify mediators linking MLKL crotonylation to autophagy-related changes, we performed MLKL IP-MS (figure 5A–D). Candidate interactors were selected for validation, and reciprocal co-immunoprecipitation confirmed an endogenous interaction between MLKL and RAB1A (figure 5E–I). RAB1A knockdown by siRNA significantly reduced p62 and p-mTOR/mTOR levels and increased LC3-II compared with the negative control group, whereas total mTOR and MLKL levels were not substantially affected (figure 5J, online supplemental figure S6). Conversely, RAB1A overexpression significantly increased p62 and p-mTOR/mTOR and reduced LC3-II, with no significant change in total mTOR or MLKL (figure 5K, online supplemental figure S7). These reciprocal results support RAB1A as a functional mediator linking upstream signals to mTOR activation and autophagy suppression in HK-2 cells. Notably, crotonylation-deficient MLKL mutants (K95R and K219R) showed reduced RAB1A GTP-binding capacity without changing total RAB1A abundance (figure 5L). To determine whether the effects of MLKL crotonylation on mTOR and autophagy markers depend on RAB1A, we knocked down RAB1A in MLKL WT-expressing cells. RAB1A knockdown in MLKL WT-expressing cells significantly reduced the p-mTOR/mTOR ratio (p=0.024), decreased p62 (p=0.006) and increased LC3-II (p=0.019) compared with the siNC control, whereas total mTOR levels were unaffected (online supplemental figure S5). Together, these findings support a model in which MLKL crotonylation is associated with activation of the RAB1A–mTOR axis and contributes to autophagy-related changes. Notably, RAB1A knockdown was performed in the MLKL WT-expressing cells; whether RAB1A specifically mediates the effects of MLKL crotonylation requires further epistasis experiments (WT vs K219R±RAB1A siRNA).

### Rapamycin partly reversed crotonylation-associated changes

HDAC1 knockdown and NaCr treatment were used to examine the broader effects of increased global crotonylation on mTOR-related signalling and autophagy markers, rather than to attribute these effects solely to MLKL crotonylation. To test whether increased global crotonylation alters autophagy-related markers, HK-2 cells were treated with NaCr. NaCr significantly increased p62/SQSTM1 and reduced LC3-II and p-AMPK/AMPK levels compared with LPS alone, whereas p-mTOR/mTOR, p-PI3K/PI3K and p-AKT/AKT were not significantly altered (figure 5M, online supplemental figure S8). Rapamycin alone markedly reduced p-PI3K/PI3K, p-AKT/AKT and p-mTOR/mTOR levels compared with the LPS group. In the NaCr+rapamycin group, p-mTOR showed a trend towards reduction compared with NaCr alone, but this did not reach statistical significance, and p-PI3K/PI3K and p-AKT/AKT were not significantly different between the NaCr and NaCr+ rapamycin groups. By contrast, the NaCr-associated decrease in p-AMPK/AMPK was significantly reversed in the NaCr+ rapamycin group, accompanied by significant restoration of autophagy-related markers, including reduced p62/SQSTM1 and increased LC3-II. These findings suggest that crotonylation-associated autophagy suppression involves AMPK inhibition and an mTOR-sensitive component, although NaCr did not significantly change p-mTOR levels. NaCr showed a trend towards increased p-mTOR that did not reach significance, without changing p-PI3K or p-AKT. Rapamycin reversed the downstream autophagy markers (p62, LC3-II) but did not significantly reduce p-mTOR in the NaCr-treated group. These observations suggest an mTOR-dependent component but do not establish direct mTOR activation by NaCr. Together with the HDAC1 knockdown data, these observations support a role for crotonylation in modulating mTOR–autophagy homeostasis. The specific contribution of individual substrates such as MLKL within this context requires further investigation.

## Discussion

LN remains one of the most serious complications of SLE and is a major driver of kidney-related morbidity and progression to kidney failure. Although current classification systems emphasise glomerular lesions, several studies have shown that tubulointerstitial inflammation and chronic damage, including interstitial fibrosis and tubular atrophy, are more closely related to long-term renal outcome than glomerular changes alone.^17 23 24^ In the present study, we identified MLKL as a differentially crotonylated protein in LN and found that increased MLKL crotonylation was associated with activation of the RAB1A–mTOR axis and altered tubular autophagy–lysosome pathway homeostasis. In renal tissues from patients with LN, we also observed increased FN and COL3 deposition, reduced LAMP1 expression and decreased LC3–LAMP1 co-localisation in tubular regions. Together, these findings support altered tubular homeostasis in LN.

One notable finding in our tissue analysis was that tubulointerstitial matrix remodelling and autophagy–lysosome pathway-related changes were detected in LN kidneys regardless of whether the biopsy was classified as class IV or class V. Although proliferative forms are generally regarded as carrying a worse renal prognosis than non-proliferative forms,^23 24^ our data suggest that tubular injury-related changes may be present across pathological subtypes. This is in line with previous studies showing that interstitial fibrosis independently predicts progression to ESKD in patients with LN.^25^ Our findings therefore support the view that glomerulus-based classification may not fully reflect the burden of tubular injury in LN. However, the small sample size may have limited our ability to detect subtype-specific differences, and a type II error cannot be excluded. These findings should be regarded as exploratory.

Autophagy is central to the maintenance of renal tubular epithelial cell integrity and inflammatory homeostasis in the kidney.^26^ In LN, autophagy has been described as a finely balanced process that may limit tissue injury by reducing cellular stress and inflammatory amplification.^13^ mTORC1 is a major signalling hub regulating autophagy and immune responses, and abnormal mTOR activation has been implicated in both SLE and LN.^14–16 27^ In the present study, several lines of evidence linked MLKL crotonylation status to altered autophagy-related markers and increased mTOR activation in tubular epithelial cells. Rapamycin partly attenuated these crotonylation-associated changes. In the cell model, autophagic flux assays using BafA1 showed that K219R-expressing cells had significantly higher LC3-II than WT cells at baseline, with comparable BafA1-induced LC3-II accumulation, consistent with increased autophagosome formation rather than impaired lysosomal degradation.^21^ These findings further support the interpretation that MLKL crotonylation is associated with suppression of autophagic activity. Although LC3-II/p62 and LC3–LAMP1 co-localisation in renal tissue do not directly measure autophagic flux,^21^ the overall pattern observed in renal tissue and cell models supports altered autophagy–lysosome pathway homeostasis in the tubular compartment in LN. We note that MLKL crotonylation at K95/K219 has not been directly demonstrated in patient kidney tissue. Obtaining fresh-frozen renal biopsy material sufficient for site-specific crotonylation analysis was not feasible due to the constraints of clinical biopsy procedures. However, the mechanistic experiments were performed in HK-2 renal tubular epithelial cells, in which MLKL crotonylation status was directly manipulated through site-directed mutagenesis. Confirmation in patient kidney tissue remains an important goal for future work.

Another important finding of this study is the implication of lysine crotonylation in LN. Previous studies have identified crotonylation as a kidney-relevant modification in both health and disease, including kidney injury and cystic kidney disease.^10 11^ In our study, PBMC crotonylome profiling revealed widespread crotonylation changes in LN and identified MLKL as a differentially crotonylated substrate. We confirmed increased crotonylation of MLKL at K95 and K219 in LN PBMCs and then linked this finding to renal tissue changes and a tubular epithelial cell mechanism. This study design allowed us to connect systemic profiling data with kidney-relevant cellular processes, while avoiding the assumption that PBMC changes directly reflect intrarenal biology.

MLKL is best known as the terminal effector of necroptosis,^28^ but it has also been implicated in inflammatory signalling and autophagy–lysosome-related processes. In this study, HK-2 cells were stimulated with LPS alone, without the TNF-α/Smac mimetic/zVAD-fmk combination typically required for canonical necroptosis induction,^28^ making full engagement of the necroptosis pathway unlikely under our experimental conditions. We performed CCK-8 and Annexin V-APC/PI assays on HK-2 cells expressing MLKL WT and the mutant constructs. In a preliminary assessment, no marked differences in cell viability or cell death were observed among groups, including under TNF-α stimulation (online supplemental figure S9), suggesting that the autophagy and mTOR phenotypes were not driven by differential cell death. Activated MLKL has been reported to translocate to intracellular membranes and impair autophagy–lysosome function,^29 30^ and necroptosis-independent roles for MLKL in the regulation of autophagy have also been described.^31 32^ MLKL activation has also been linked to inflammasome signalling and cytokine maturation,^33^ as well as MAPK/PI3K-associated inflammatory pathways.^34^ In our tubular epithelial cell model, crotonylation-deficient MLKL mutants were associated with a more favourable autophagy-related marker profile and lower cytokine production, whereas the charge-neutralising K219Q mutant showed the opposite trend. Because K-to-Q substitution does not fully reproduce the chemical and steric features of true crotonylation, we interpret K219Q as a putative acylation-related variant rather than a definitive crotonylation mimic. Accordingly, results derived from K219Q should be regarded as hypothesis-generating. Even so, the concordant findings obtained with HDAC1 knockdown and sodium crotonate treatment support a link between increased crotonylation states and suppression of autophagy-related markers. HDAC1 knockdown and NaCr treatment were used to examine the general effects of increased crotonylation on mTOR signalling and autophagy markers, not to establish that these effects act exclusively through MLKL. Although HDAC1 knockdown increased MLKL crotonylation specifically, the concurrent global acylation changes mean that the downstream effects cannot be attributed to MLKL alone. Rapamycin was used to test whether the mTOR pathway is involved in crotonylation-associated autophagy suppression and should be interpreted as evidence of an mTOR-dependent component rather than a MLKL-specific mechanism.

Mechanistically, we identified RAB1A as a prominent MLKL interactor and found that MLKL crotonylation increased the GTP-binding capacity of RAB1A without altering total RAB1A abundance. RAB1A is classically involved in ER-to-Golgi trafficking,^35^ but it has also been described as an upstream regulator of mTORC1 through its effects on Rheb–mTORC1 interactions and nutrient signalling.^22 36^ Our data therefore support a model in which MLKL crotonylation is associated with activation of a RAB1A–mTOR signalling axis and with changes in autophagy-related markers in tubular epithelial cells. In line with this model, RAB1A knockdown in MLKL WT-expressing cells reduced mTOR activation and partly reversed the autophagy-suppressive marker changes, providing further evidence that RAB1A contributes to the mTOR-related effects in these cells. However, a full epistasis design (WT vs K219R±RAB1A siRNA) would be needed to confirm that these effects are specifically mediated through RAB1A.

From a translational perspective, these findings point to a potentially targetable pathway. Sodium crotonate increased p62 and decreased LC3-II, and rapamycin partly reversed these changes, although p-mTOR was not significantly altered by NaCr. The sensitivity of p62 and LC3-II to rapamycin supports an mTOR-dependent component, and this pathway warrants further study in LN-related tubular injury.

This study has several limitations. First, MLKL crotonylation at K95/K219 was identified and validated in PBMCs but has not been directly shown in patient kidney tissue. Although the mechanistic experiments were performed in HK-2 renal tubular epithelial cells, confirmation that MLKL is crotonylated at these sites in the tubular compartment in vivo is needed in future studies. Second, in renal biopsy tissues, reduced LAMP1 expression and decreased LC3–LAMP1 co-localisation support altered autophagy–lysosome pathway homeostasis, but LC3 signal did not differ significantly and direct flux assays were not performed in tissue. In the cell model, BafA1-based flux assays showed that K219R-expressing cells had higher LC3-II levels than WT both before and after lysosomal inhibition, with comparable ΔLC3-II, consistent with increased autophagosome formation rather than a degradation block. However, the flux assay was performed only for K219R; whether K95R shows a similar pattern remains to be confirmed. The class IV vs class V comparison was underpowered, and the possibility of a type II error should be acknowledged. Third, K-to-R substitutions remove not only crotonylation but also other lysine modifications, K-to-Q substitutions do not reproduce crotonylation chemistry, and both K95 and K219 lie within functionally important MLKL domains. HDAC1 knockdown and NaCr treatment alter global acylation and cannot attribute downstream effects specifically to MLKL; these tools were used to examine the broader crotonylation–mTOR relationship, and future epistasis experiments are needed to isolate the contribution of MLKL crotonylation. Fourth, LPS stimulation models innate immune-mediated tubular injury but does not fully recapitulate the immune-complex- and interferon-driven injury of LN; validation with LN-relevant stimuli is an important next step. In addition, control kidney tissues were obtained from tumour-nephrectomy specimens; although renal cortex was sampled at least 5 cm from the tumour margin, these tissues may not fully represent normal tubular biology, and no patients were receiving mTOR inhibitors at the time of sampling.

In summary, this study integrates PBMC crotonylome profiling, renal tubulointerstitial tissue analysis and mechanistic experiments in tubular epithelial cells to show that MLKL crotonylation is associated with RAB1A–mTOR signalling and altered autophagy–lysosome pathway homeostasis in LN (figure 5N). Our findings also suggest that tubular injury-related changes may be shared across pathological subtypes and may not be fully reflected by glomerulus-based classification alone.

## Supplementary material

10.1136/lupus-2026-002116 online supplemental figure 1

10.1136/lupus-2026-002116 online supplemental figure 2

10.1136/lupus-2026-002116 online supplemental figure 3

10.1136/lupus-2026-002116 online supplemental figure 4

10.1136/lupus-2026-002116 online supplemental figure 5

10.1136/lupus-2026-002116 online supplemental figure 6

10.1136/lupus-2026-002116 online supplemental figure 7

10.1136/lupus-2026-002116 online supplemental figure 8

10.1136/lupus-2026-002116 online supplemental figure 9

10.1136/lupus-2026-002116 online supplemental figure 10

10.1136/lupus-2026-002116 online supplemental table 1

10.1136/lupus-2026-002116 online supplemental table 2

## Data Availability

Data are available upon reasonable request.
