# Supplementary material for: Crotonylome profiling identifies MLKL crotonylation in lupus nephritis associated with RAB1A–mTOR signalling and autophagy changes in tubular epithelial cells
Source: Lupus Sci Med. 2026 Aug 31;13(2):e002116. doi: 10.1136/lupus-2026-002116 (PMC13536117; doi:10.1136/lupus-2026-002116)

Full unedited gel for Figure 1

Figure 1A

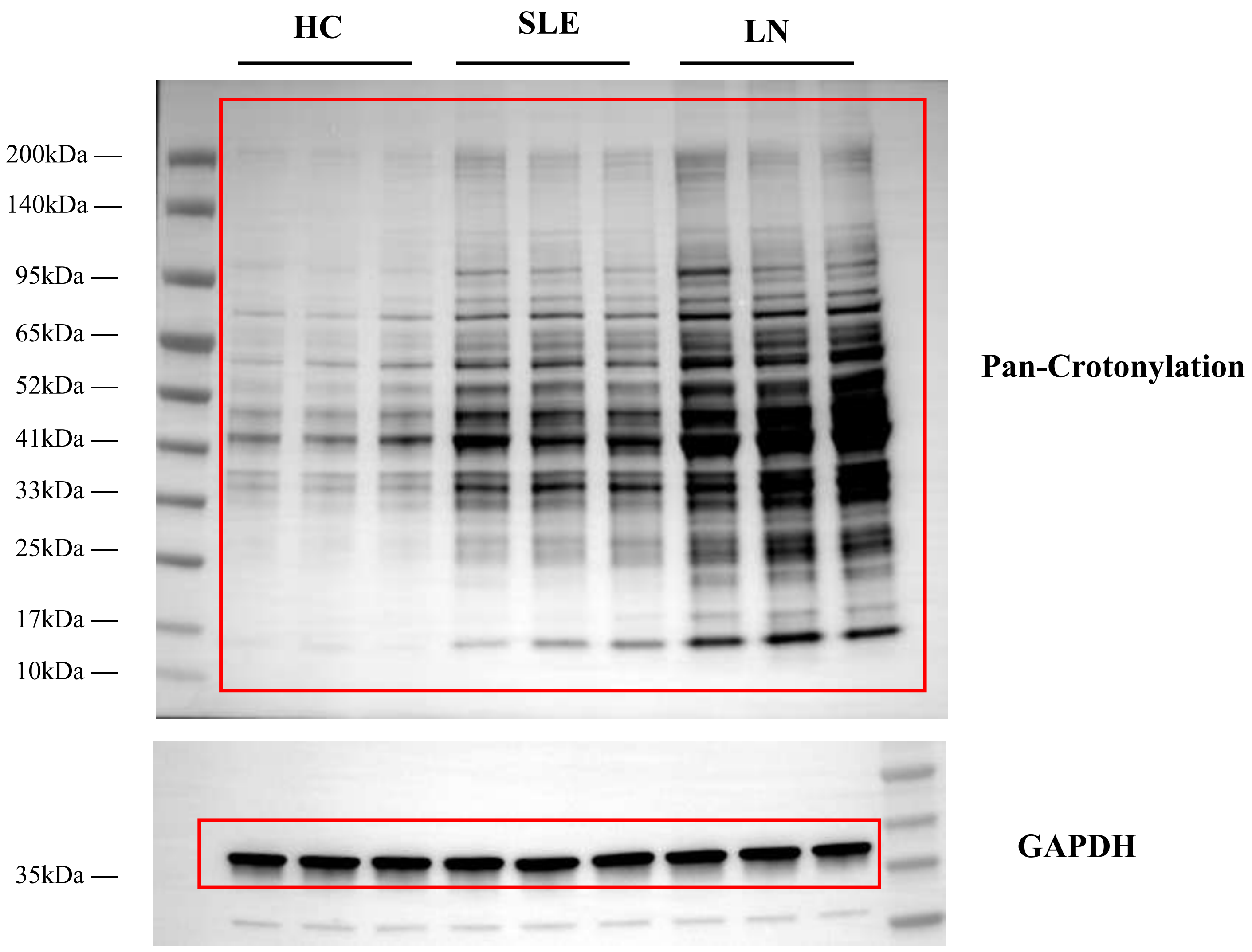

Full unedited gel for Figure 2

Figure 2C

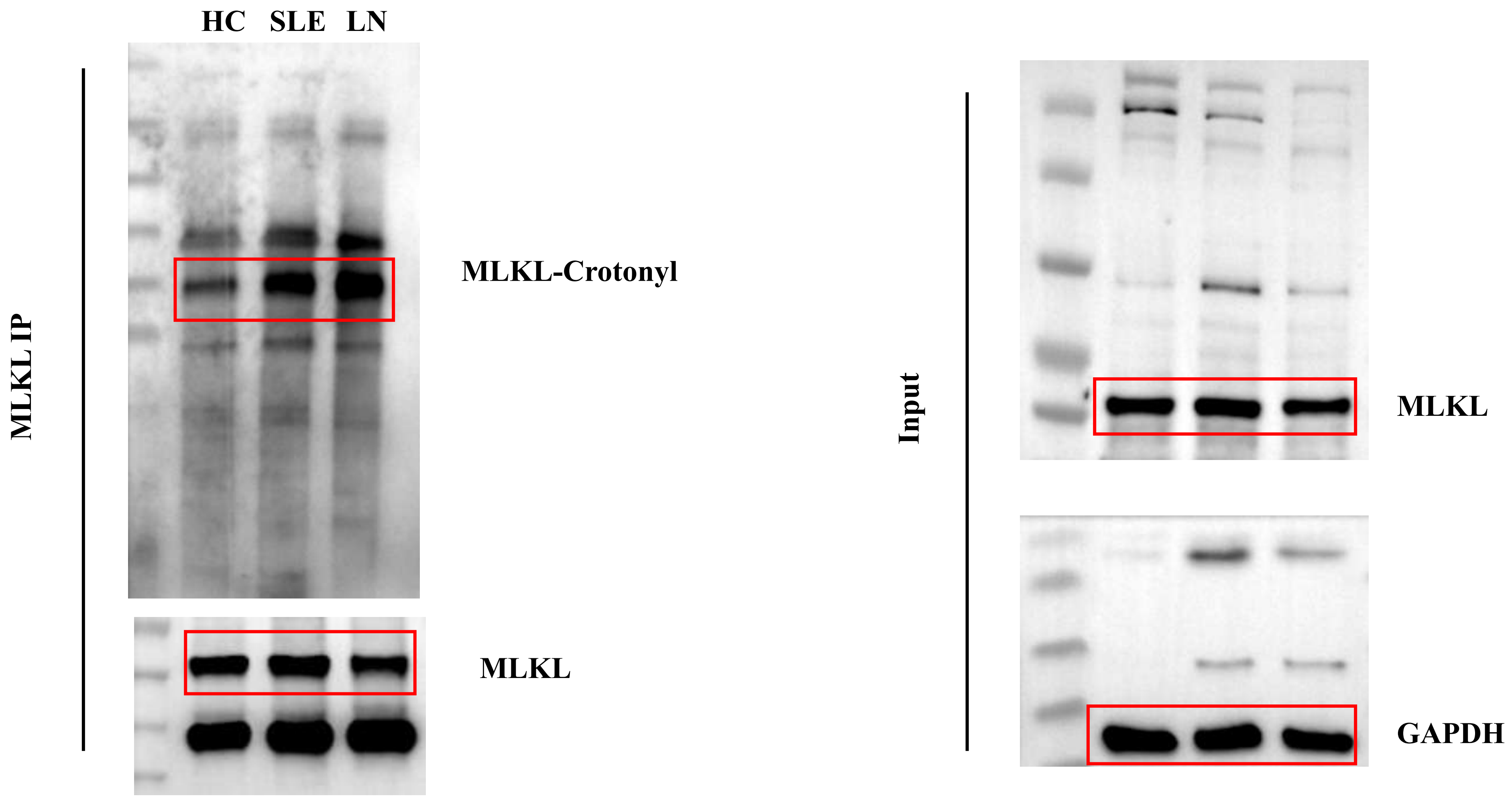

Full unedited gel for Figure 4

Figure 4A

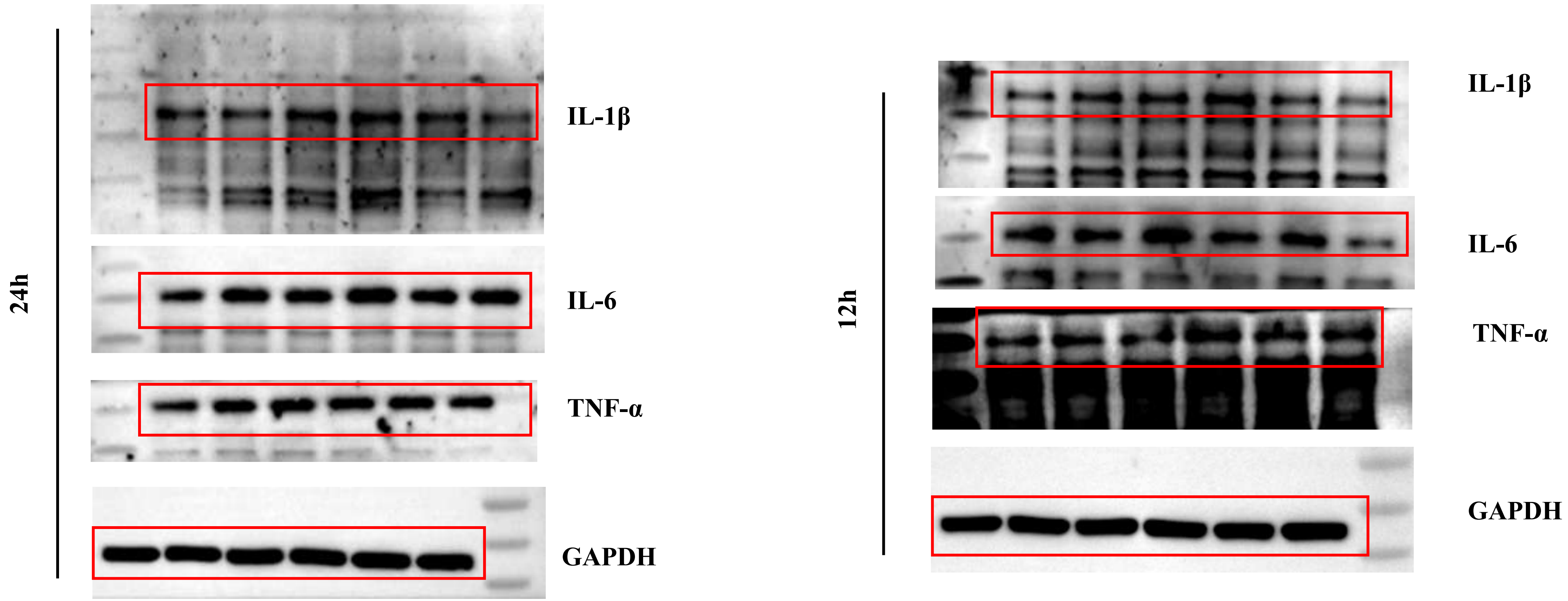

Full unedited gel for Figure 4

Figure 4B

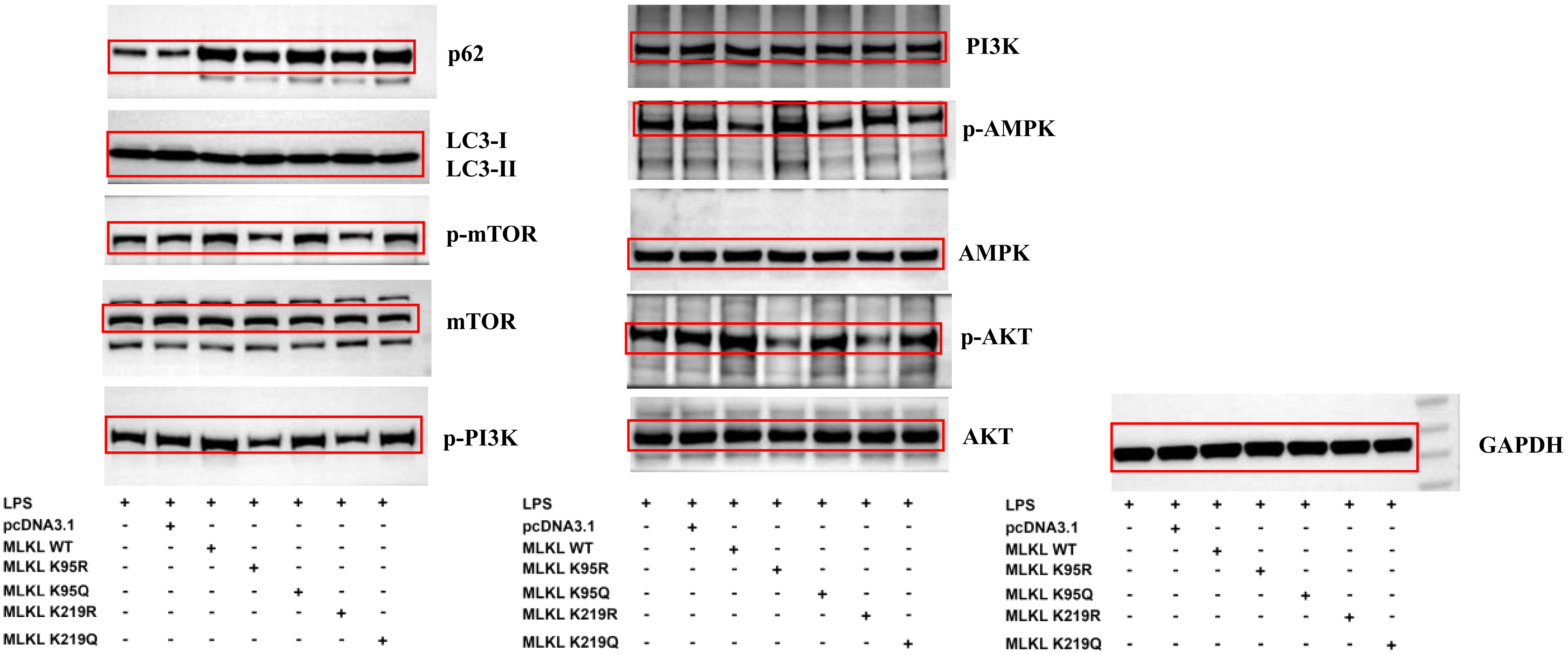

Full unedited gel for Figure 4

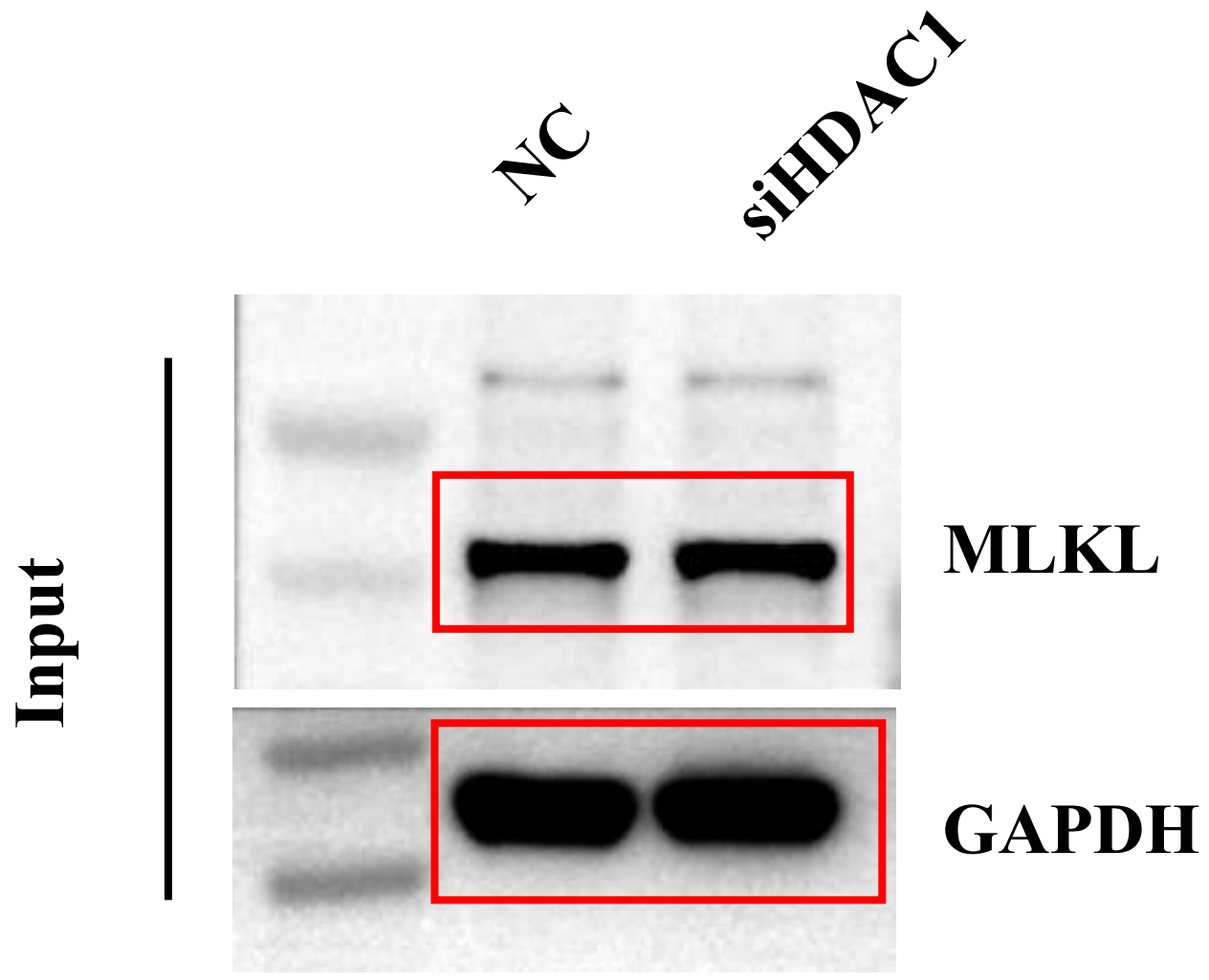

Figure 4F

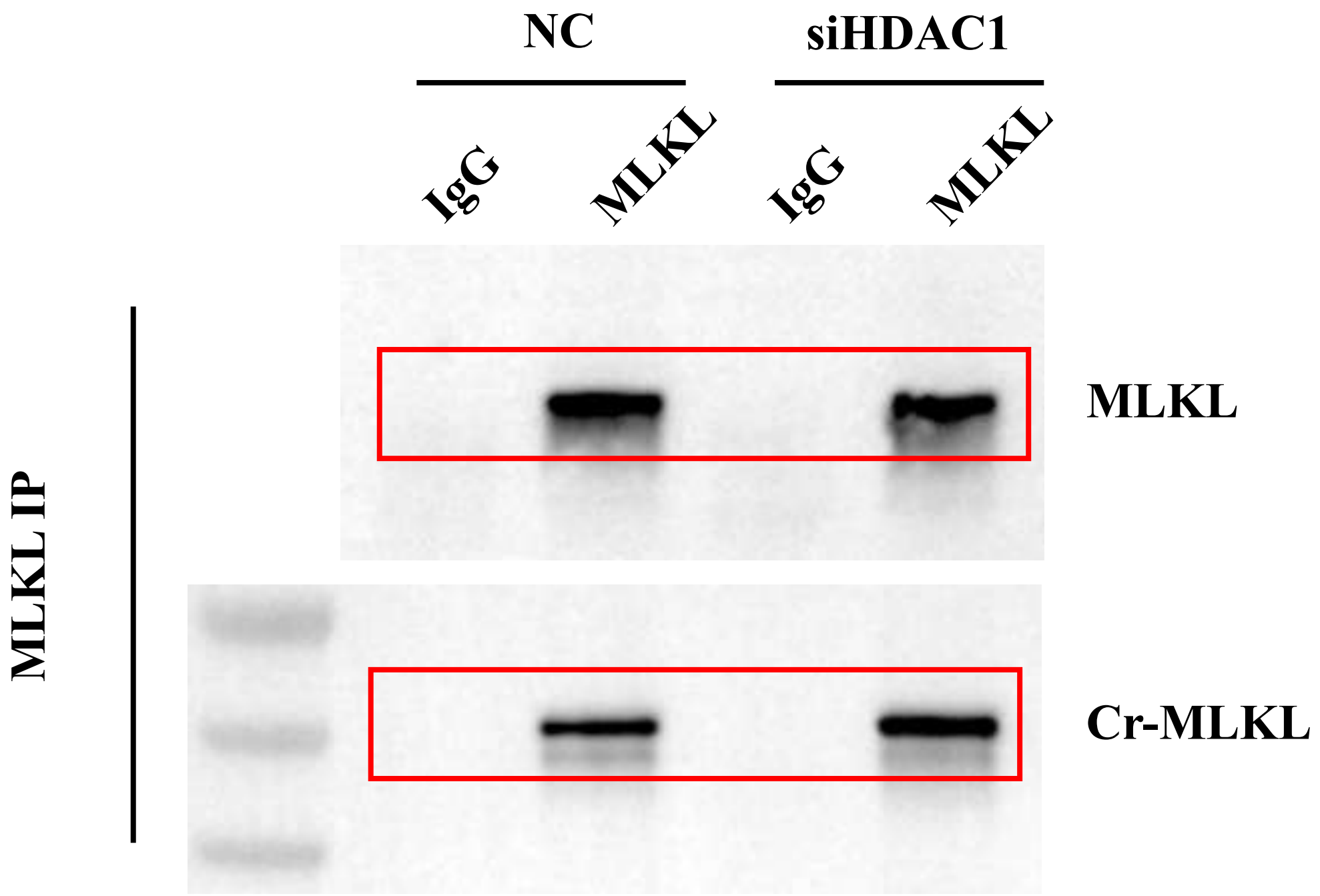

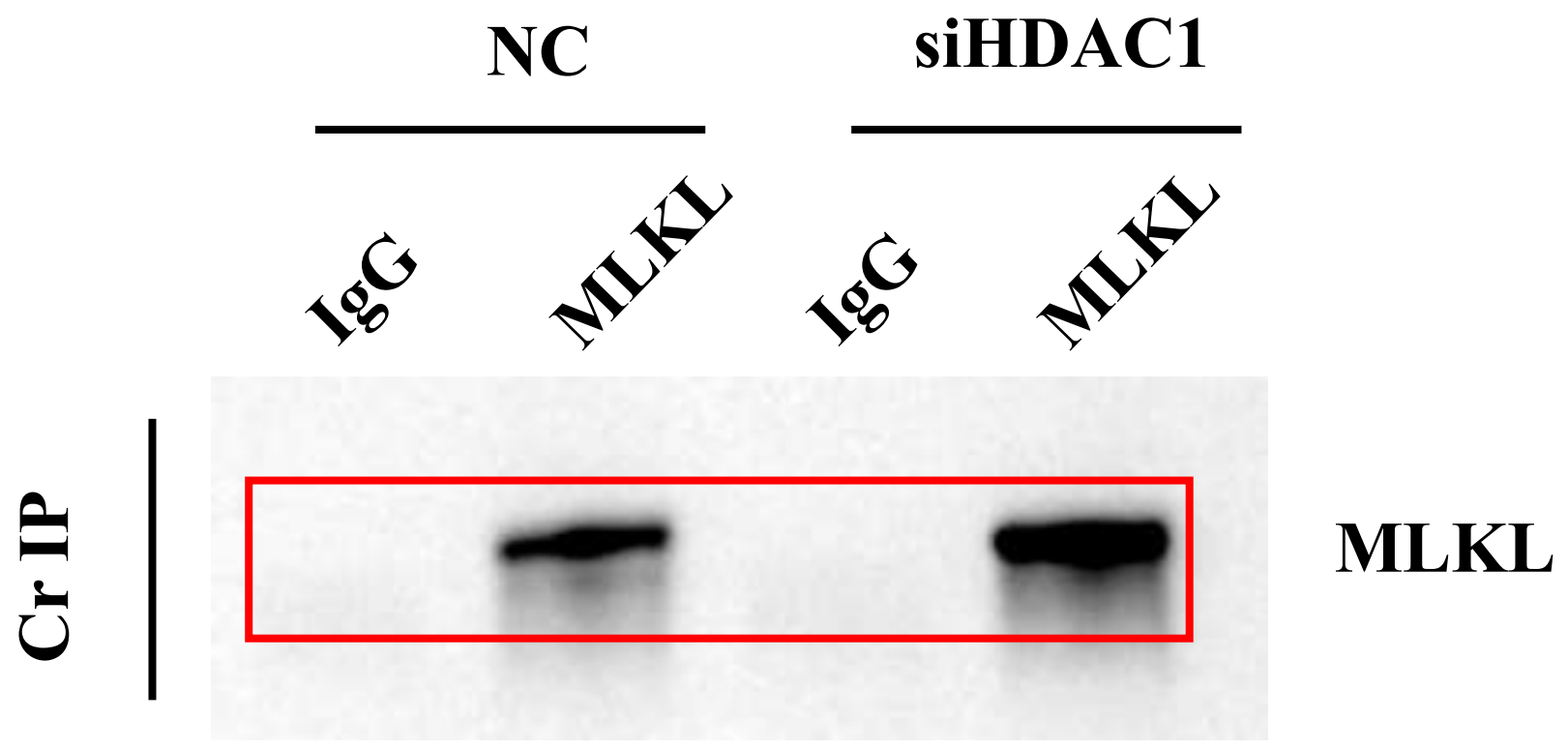

Full unedited gel for Figure 4

Figure 4G

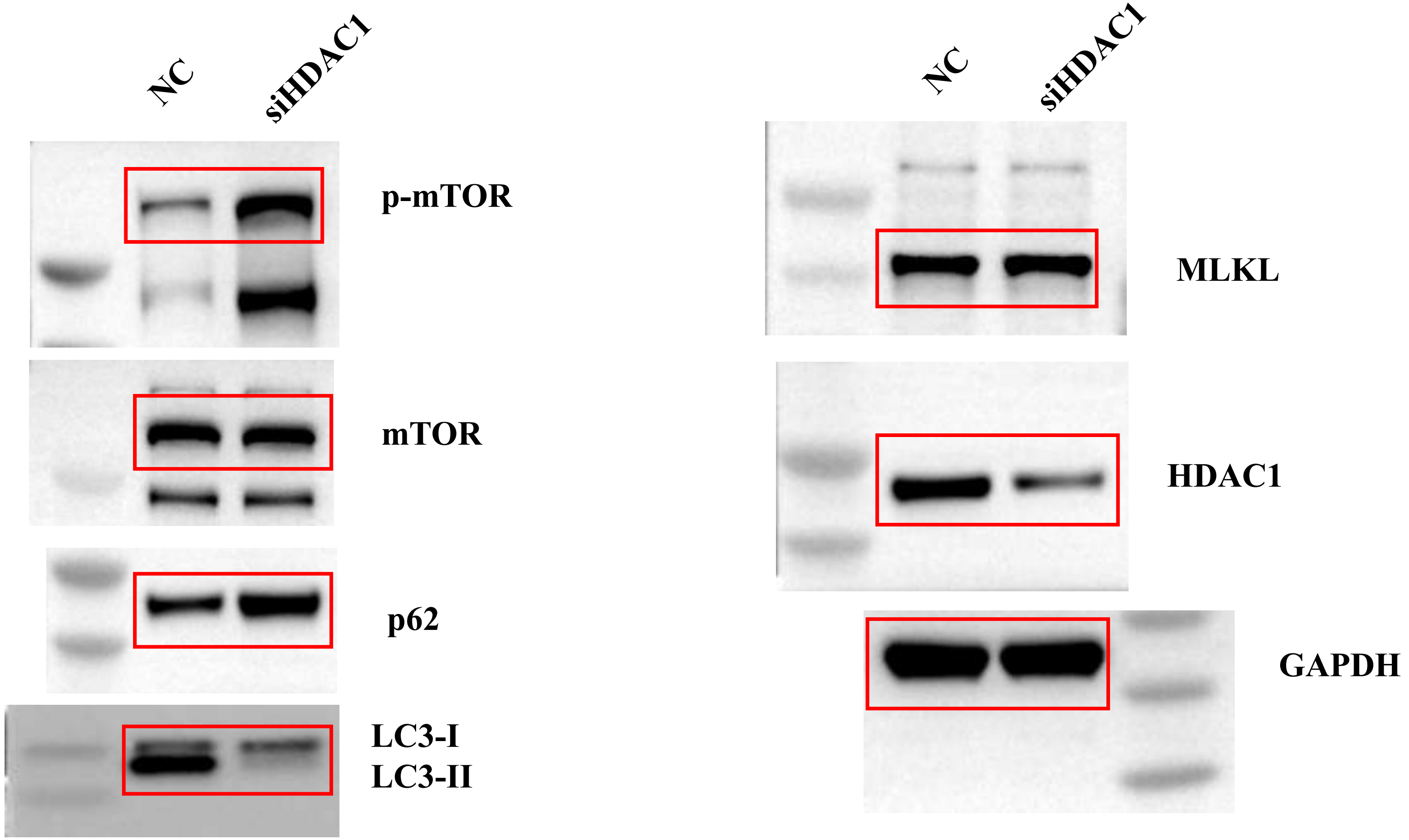

Full unedited gel for Figure 5

Figure 5B

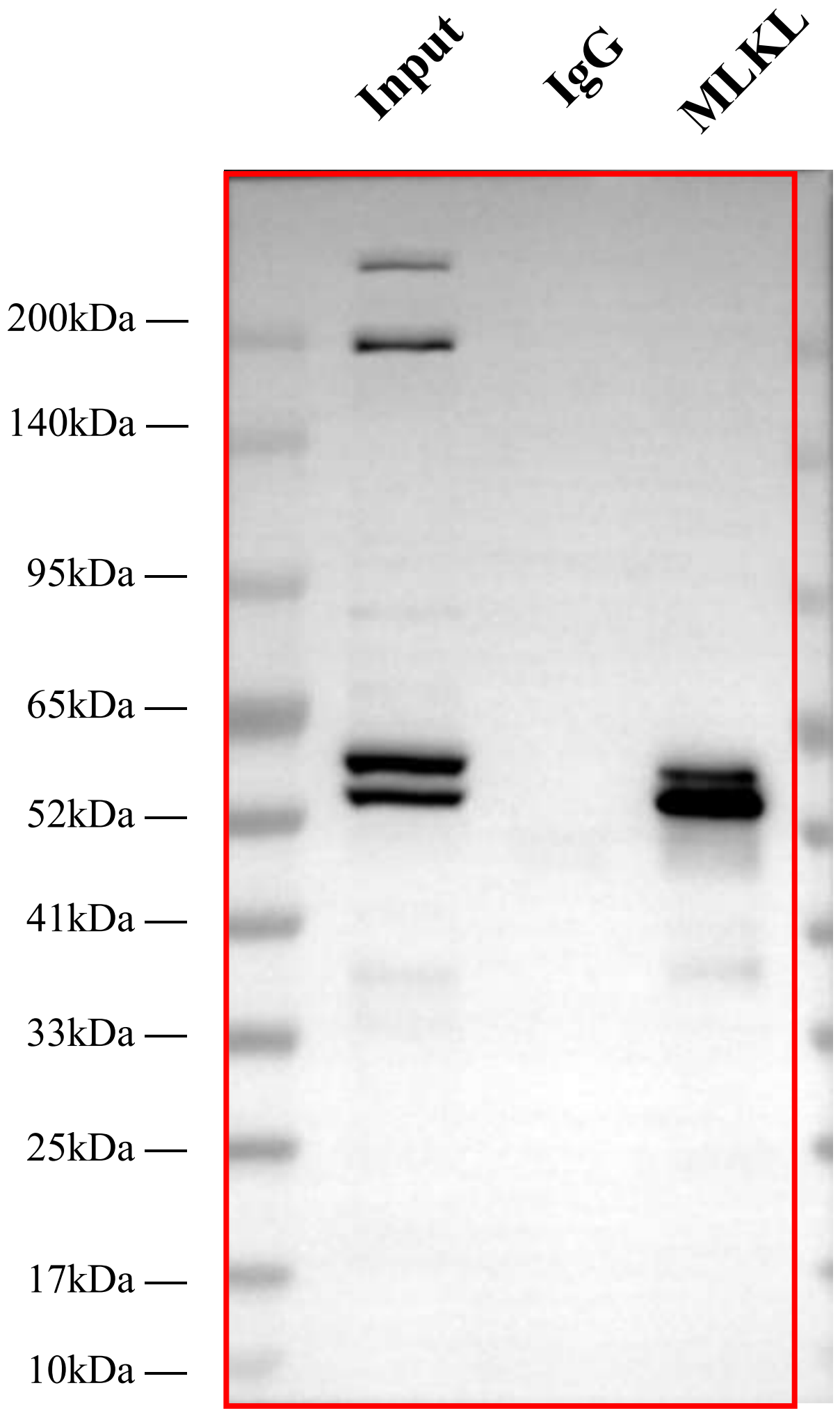

Figure 5E

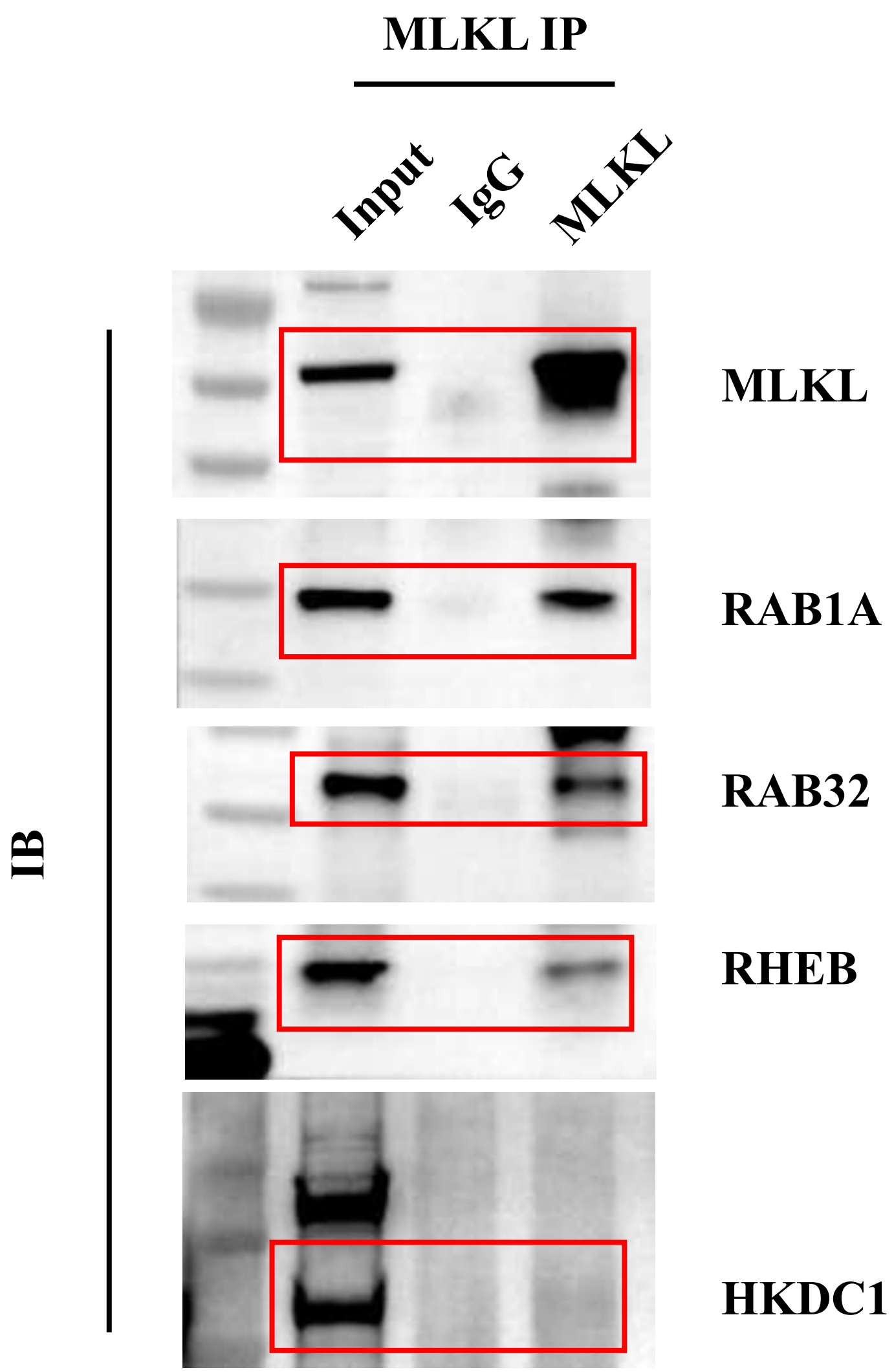

Full unedited gel for Figure 5

Figure 5F

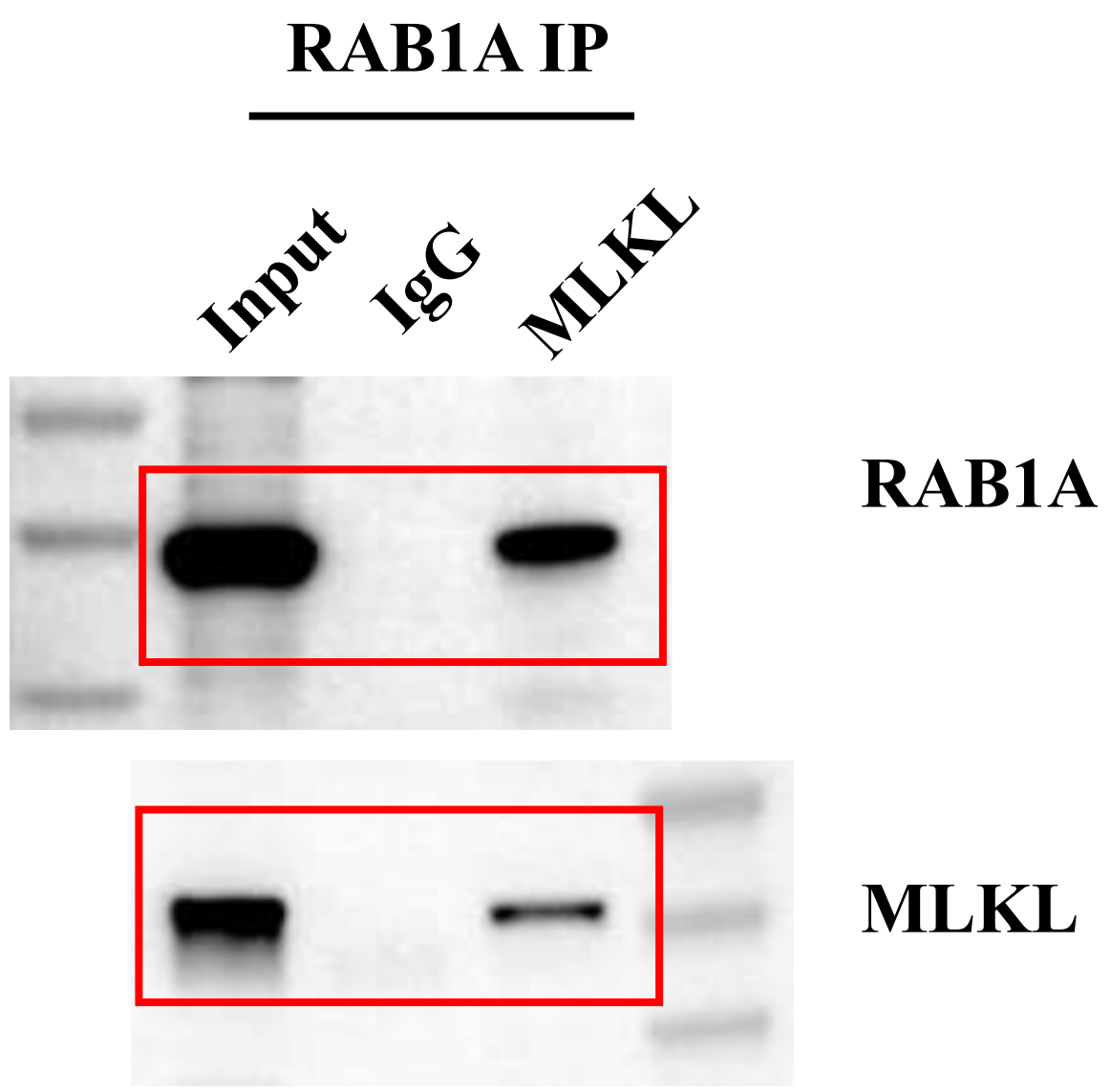

Figure 5G

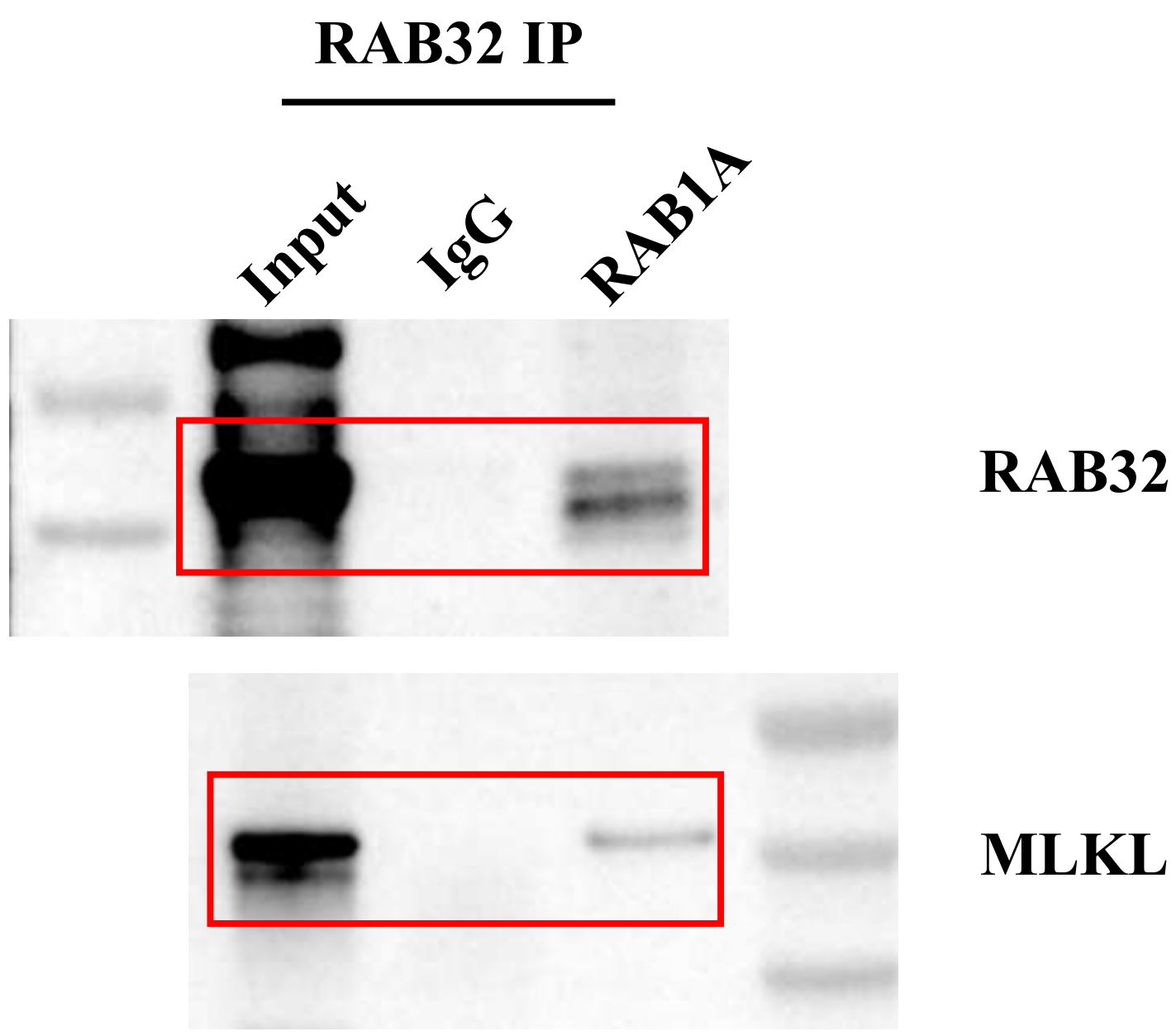

Full unedited gel for Figure 5

Figure 5H

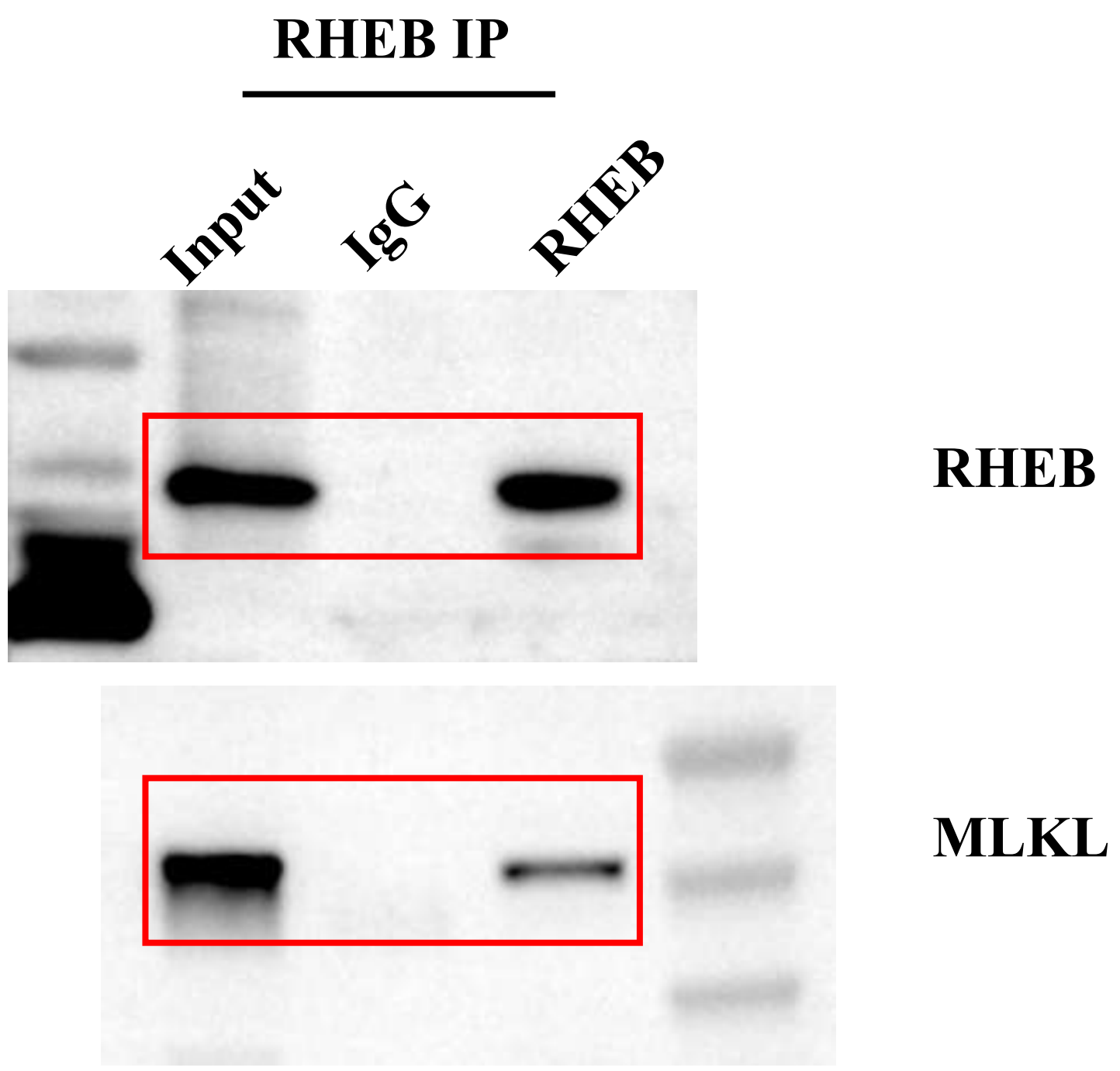

Figure 5I

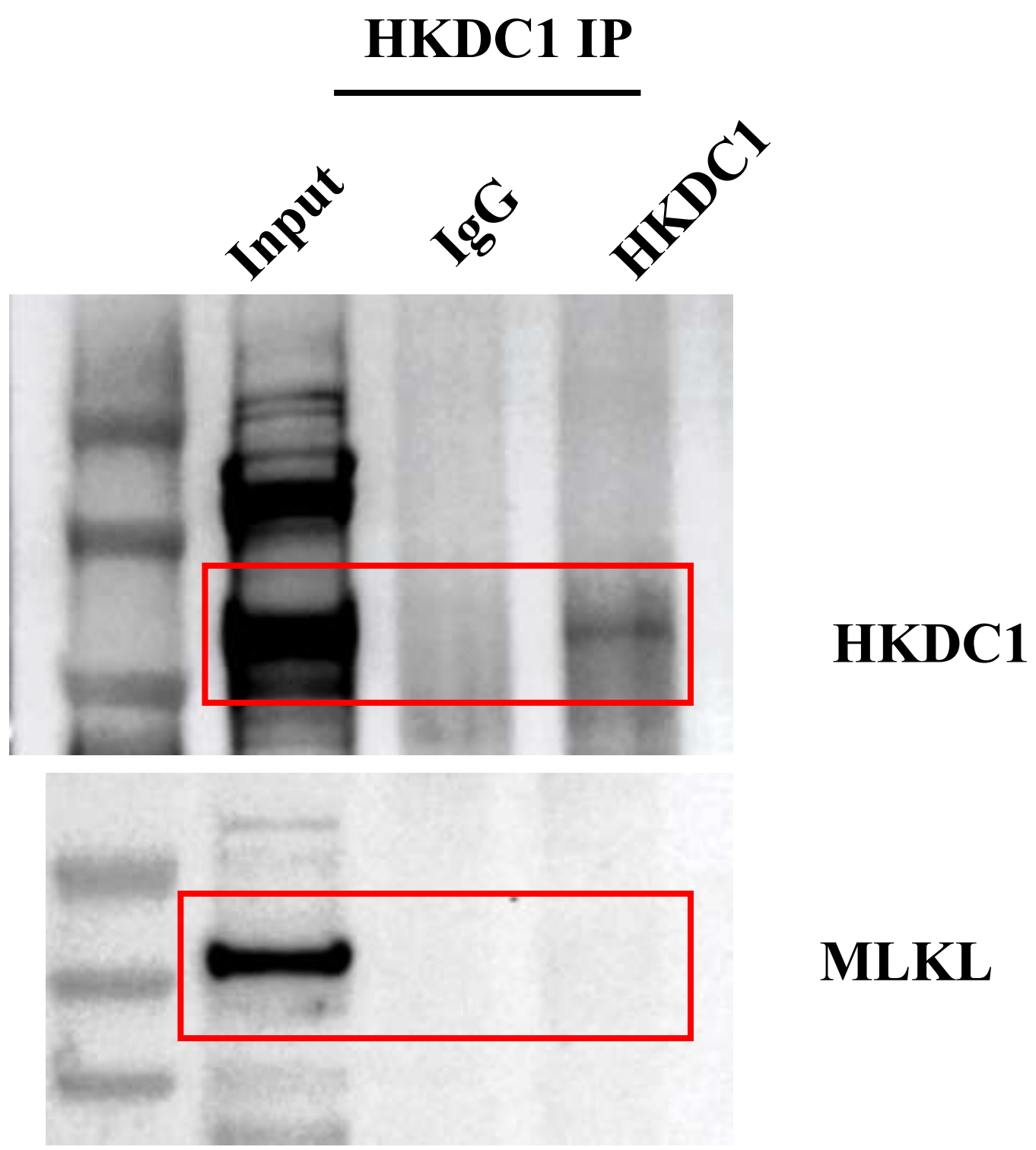

Full unedited gel for Figure 5

Figure 5J

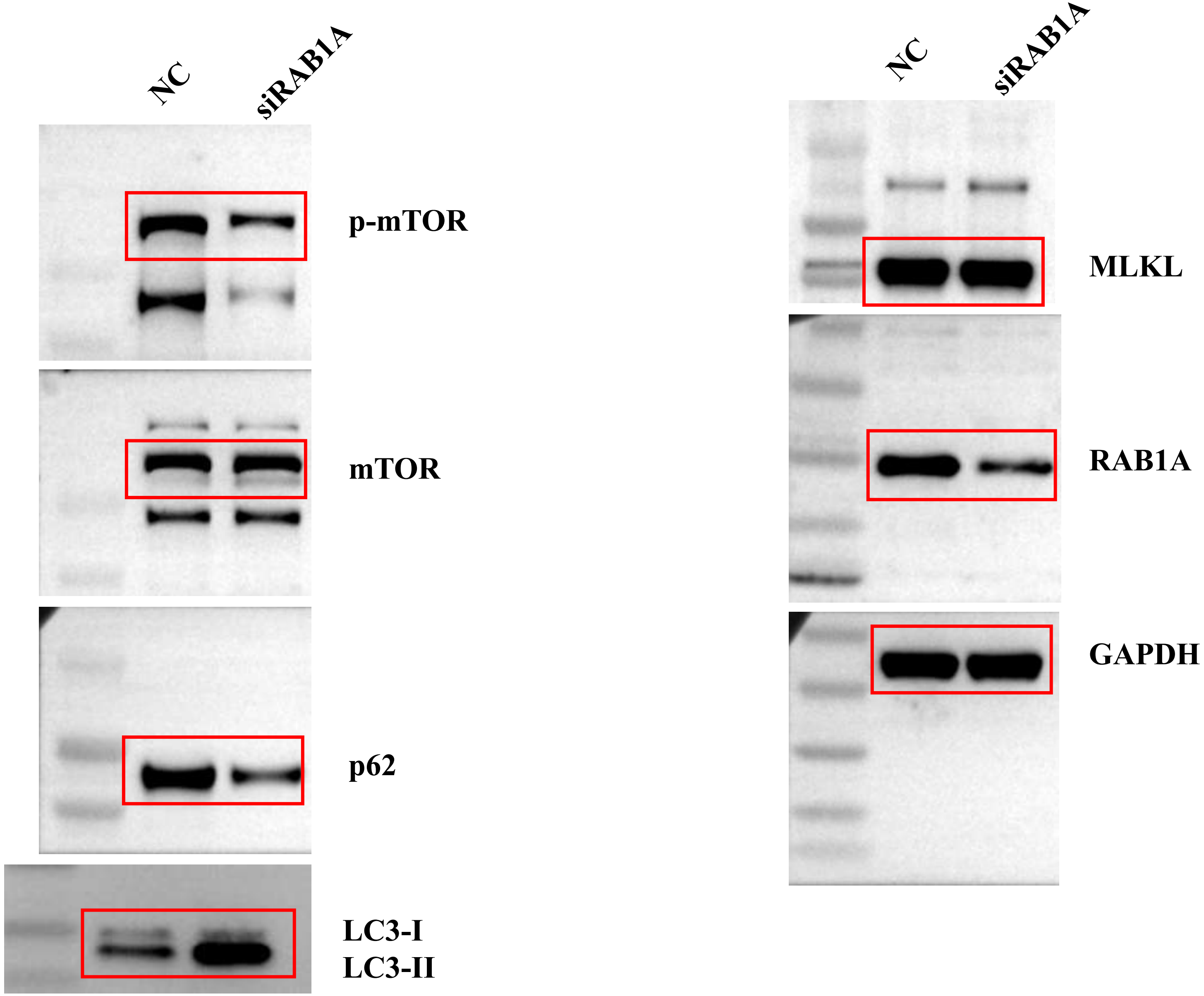

Full unedited gel for Figure 5

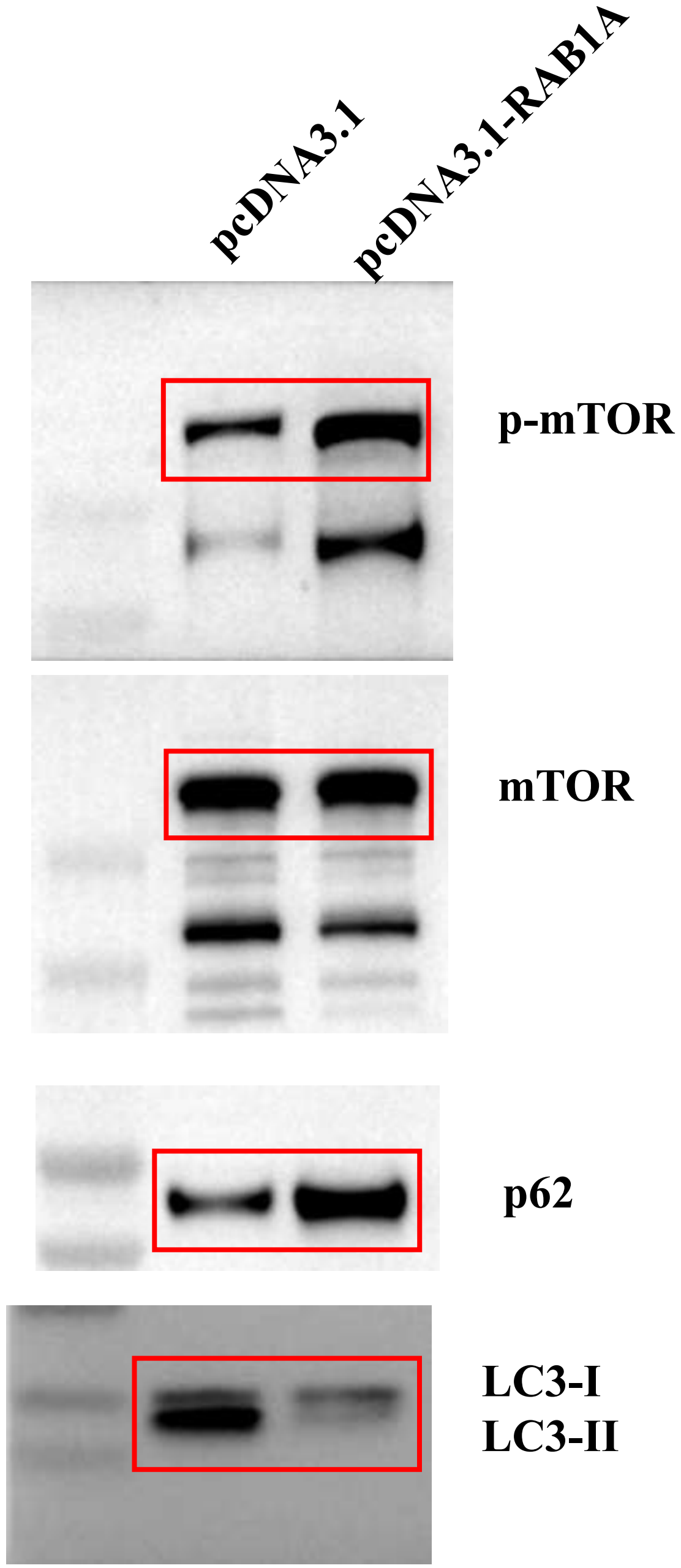

Figure 5K

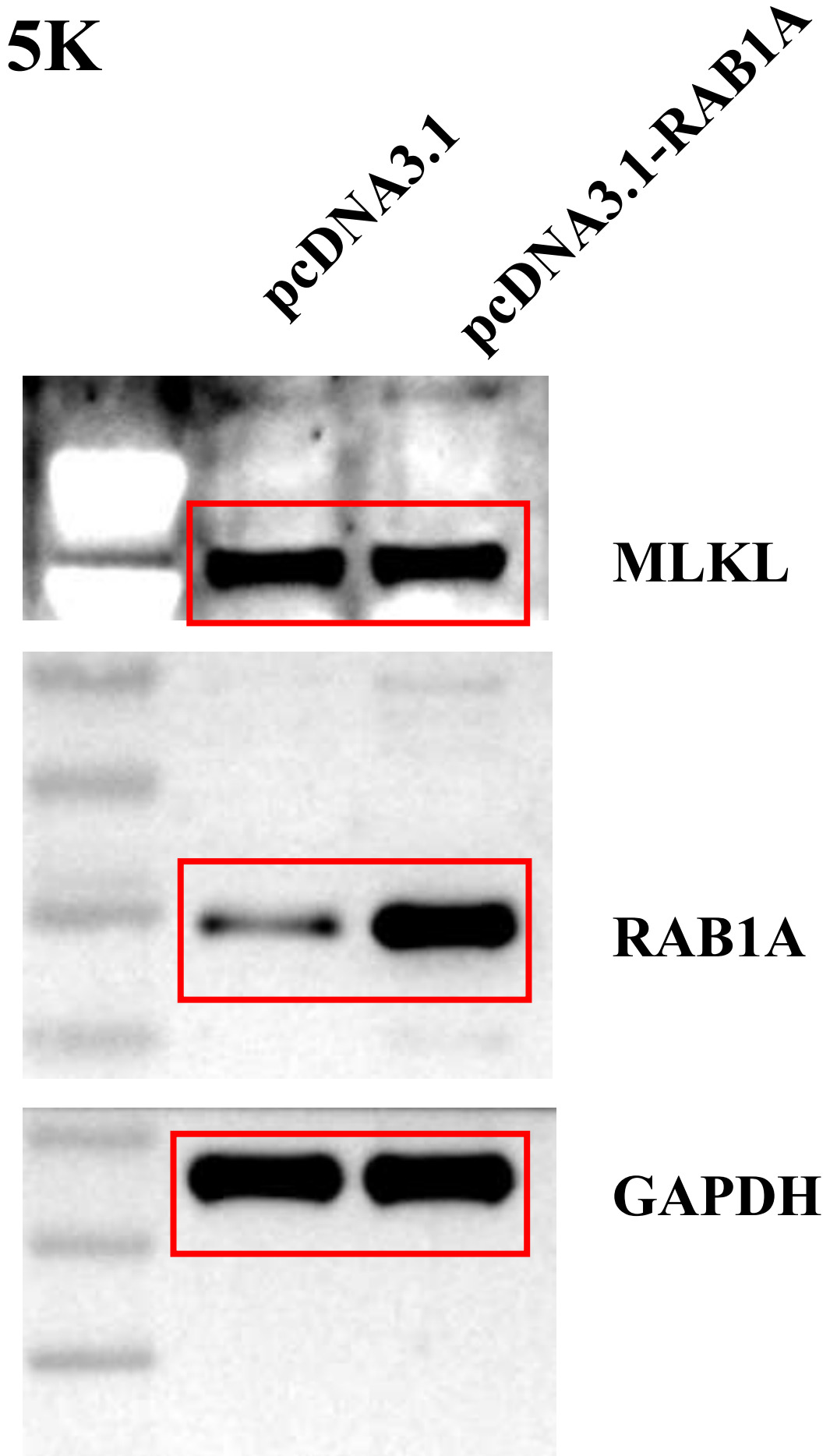

Full unedited gel for Figure 5

Figure 5L

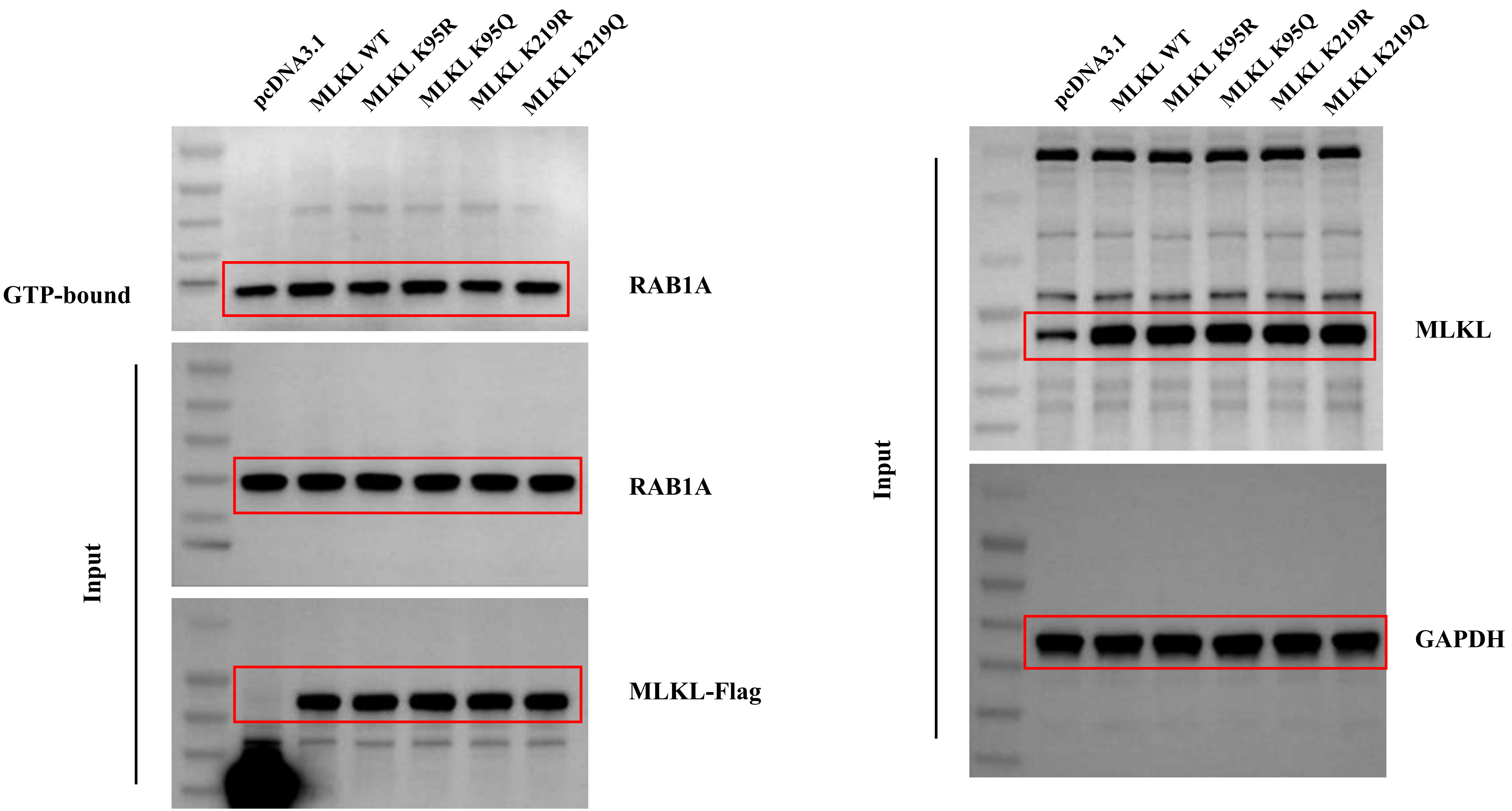

Full unedited gel for Figure 5

Figure 5M

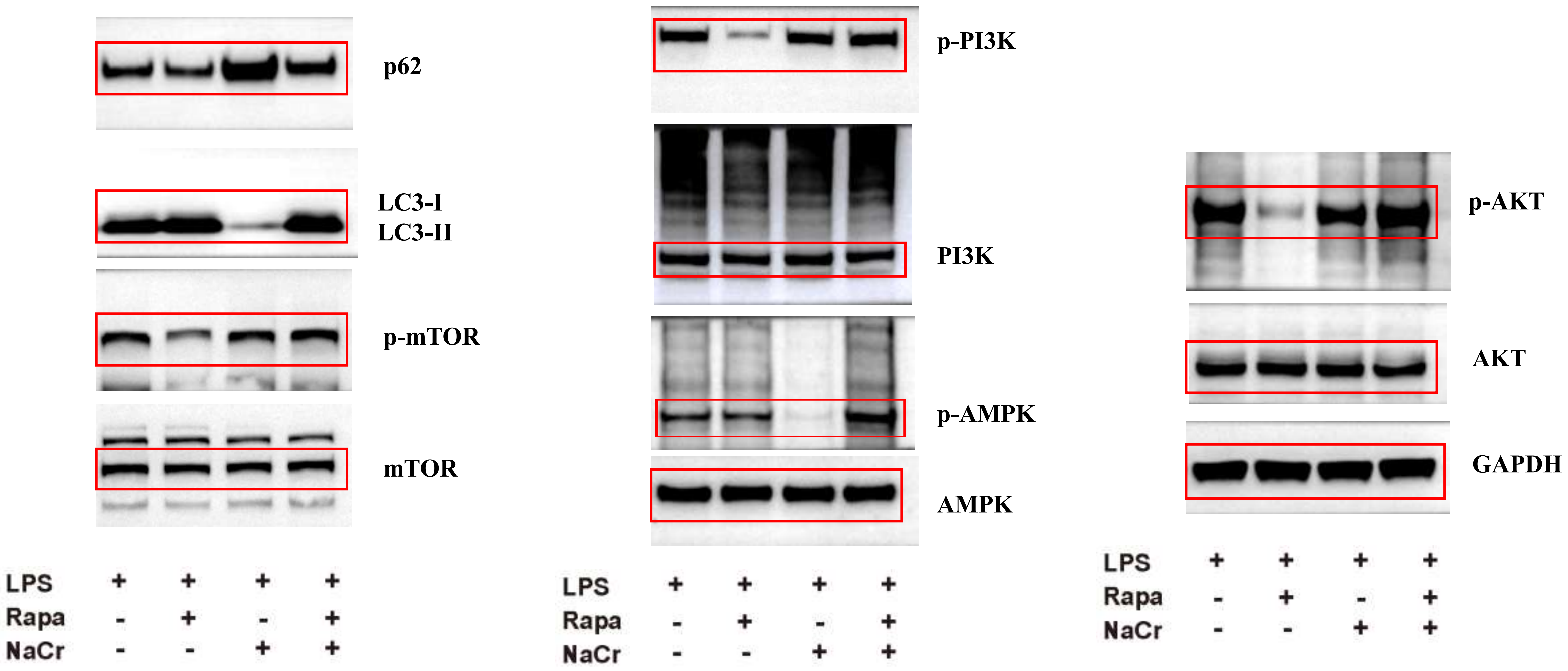

Full unedited gel for Figure S4

Figure S4A

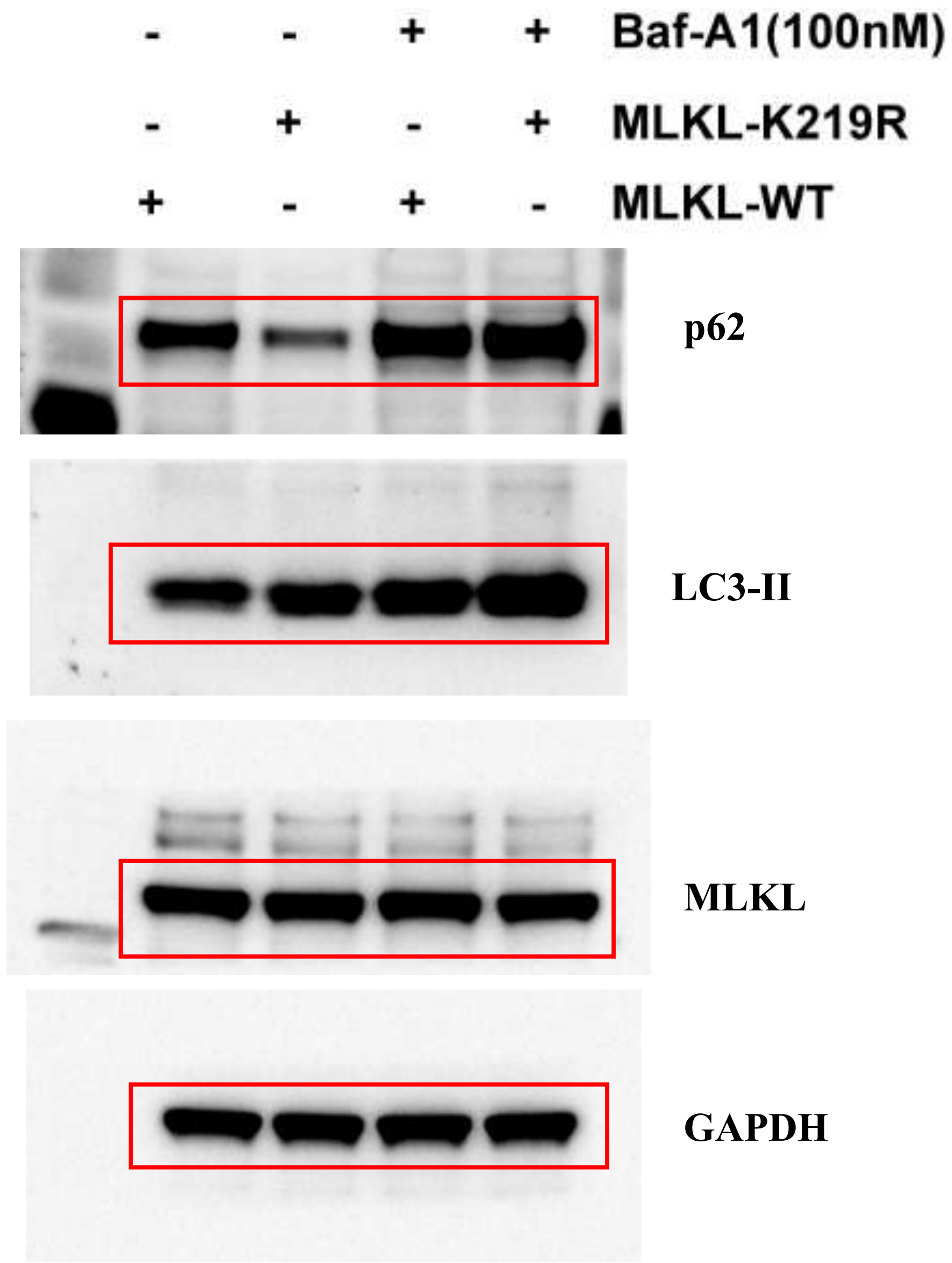

Full unedited gel for Figure S5

Figure S5A

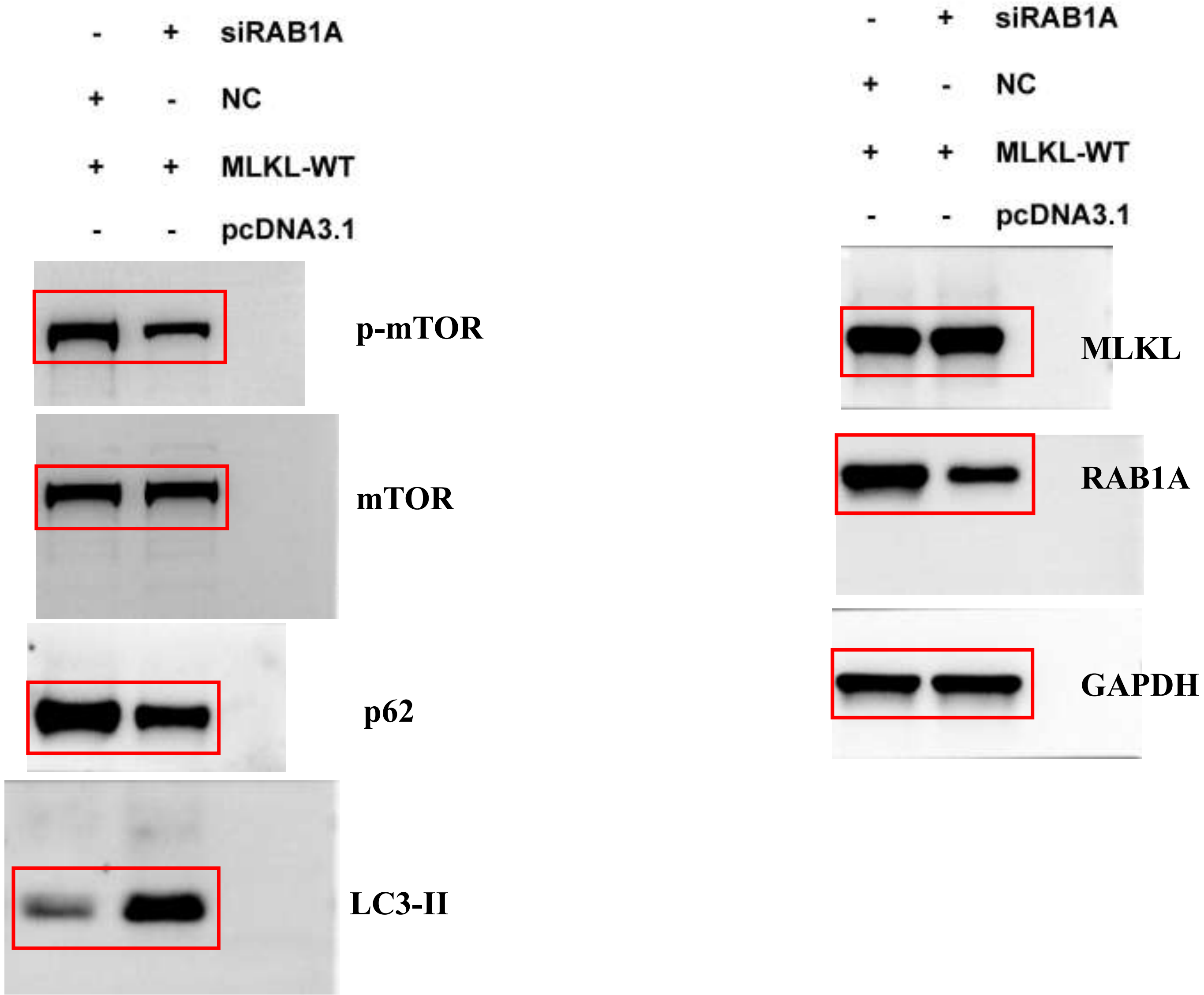

Supplement: Supplementary file 1 [file lupus-13-2-s010.pdf]
